# Moisture-Electric–Moisture-Sensitive Heterostructure Triggered Proton Hopping for Quality-Enhancing Moist-Electric Generator

**DOI:** 10.1007/s40820-023-01260-w

**Published:** 2023-12-18

**Authors:** Ya’nan Yang, Jiaqi Wang, Zhe Wang, Changxiang Shao, Yuyang Han, Ying Wang, Xiaoting Liu, Xiaotong Sun, Liru Wang, Yuanyuan Li, Qiang Guo, Wenpeng Wu, Nan Chen, Liangti Qu

**Affiliations:** 1 Key Laboratory of Cluster Science, Ministry of Education of China, Key Laboratory of Photoelectronic/Electrophotonic Conversion Materials, School of Chemistry and Chemical Engineering, Beijing Institute of Technology, Beijing, 100081 People’s Republic of China; 2 Yangtze Delta Region Academy of Beijing Institute of Technology, Jiaxing, 314019 People’s Republic of China; 3 Department of Chemistry, Key Laboratory of Organic Optoelectronics & Molecular Engineering, Ministry of Education, Tsinghua University, Beijing, 100084 People’s Republic of China

**Keywords:** Moist-electric generators, Grotthuss proton hopping, Fast response, Durable electrical output, Personal health monitoring

## Abstract

Moisture-enabled electricity (ME) is a method of converting the potential energy of water in the external environment into electrical energy through the interaction of functional materials with water molecules and can be directly applied to energy harvesting and signal expression. However, ME can be unreliable in numerous applications due to its sluggish response to moisture, thus sacrificing the value of fast energy harvesting and highly accurate information representation. Here, by constructing a moisture-electric–moisture-sensitive (ME-MS) heterostructure, we develop an efficient ME generator with ultra-fast electric response to moisture achieved by triggering Grotthuss protons hopping in the sensitized ZnO, which modulates the heterostructure built-in interfacial potential, enables quick response (0.435 s), an unprecedented ultra-fast response rate of 972.4 mV s^−1^, and a durable electrical signal output for 8 h without any attenuation. Our research provides an efficient way to generate electricity and important insight for a deeper understanding of the mechanisms of moisture-generated carrier migration in ME generator, which has a more comprehensive working scene and can serve as a typical model for human health monitoring and smart medical electronics design.

**Supplementary Information:**

The online version contains supplementary material available at 10.1007/s40820-023-01260-w.

## Highlights

- An efficient moist-electric generator with ultra-fast electric response to moisture is achieved by triggering Grotthuss protons hopping in the moisture-electric–moisture-sensitive heterostructure.
- The moist-electric generator produces a quick response (0.435 s), an unprecedented ultra-fast response rate of 972.4 mV s^−1^ to alternating moisture stimulation and stable output for 8 h.
- An obstructive sleep apnea hypoventilation syndrome diagnostic system based on a moist-electric generator was developed to monitor hypopnea and apnea in real time and successfully diagnose them with early warning.

**Supplementary Information:**

The online version contains supplementary material available at 10.1007/s40820-023-01260-w.

## Graphical abstract

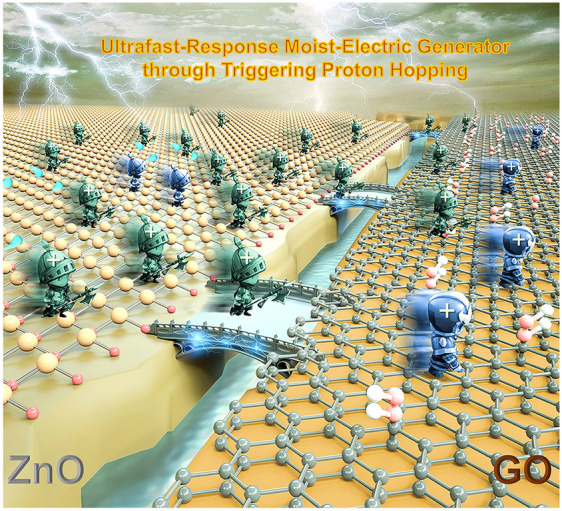

## Introduction

Human intelligent electronic devices with various functions, such as navigation [1], exercise [2], environmental monitoring [3], physiology, and health monitoring [4–6] have flourished to meet the diverse needs of our daily life. In recent years, the emergence of new energy sources such as thermoelectricity [7–9], piezoelectricity [10–12] and triboelectricity [13–16] has directly converted potential energy in the human body or the nearby environment into valuable electrical signals for detection and information expression, forming self-powered intelligent device that integrates energy harvesting and efficient signal expression and promoting the development of intelligent electronic equipment. Covering 71% of the Earth's surface area, water is not only widely distributed in lakes, oceans, soil, natural evaporation and human respiration, but also is the largest energy carrier on Earth [17]. In this regard, ME generated by interacting functional materials with water molecules is an advanced technology for extracting electrical energy directly from water and converting it into directly applicable signals [18]. However, there are still many problems/shortcomings in the application of ME materials that use the diffusion potential energy of water molecules or spontaneous diffusion driven by ion concentration gradients to bring electrical signal response in high-precision human intelligent devices, such as slow response rate (less than 0.1 V s^−1^) and short lifetime of induced electrical signals.

The key to developing fast and efficient energy harvesting from the environment is the rational design of hydroelectric materials. Although some progress has been made in the development of new moist-electric materials, such as graphene oxide [19–29], polymers [30–46], carbon materials [47–54], etc., these reported moist-electric devices only rely on carrier diffusion in a single component to generate electricity, so most of these can only output pulsed electrical signals that change synchronously with humidity, while a few ME generators can continuously output electrical energy but respond very slowly to moisture [55–58]. A ME generator capable of both fast response to moisture and long-term stable output characteristics has yet to be realized. So effective compounding and microstructure construction of functional structures are needed to compose fast and efficient moist-electric material/device systems. Semiconductor-like materials typically have carrier concentrations that are more than three orders of magnitude lower relative to metallic materials, which makes them responsive to external source stimuli imposed by various physical fields such as light, electricity, and heat [59–61]. It is worth mentioning that semiconductor materials also include a class of moisture-sensitive semiconductors, which are semiconductor materials whose moisture-sensitive effects. With the assistance of external electrical power, moisture-sensitive semiconductor materials can use the change in conductivity or work function caused by the adsorption of moisture (water vapor) on the surface to affect the expression of electrical signals. These special properties allow moisture-sensitive semiconductors to show unique superiority in some energy devices. Inspired by our previous work [62], we designed a novel bilayer heterostructure, which combines the advantages of both moisture-sensitive materials that are sensitive to moisture and ME materials capable of harvesting energy from moisture, achieving ultra-fast response speed and sustained power output previously unattainable with a single ME generator.

**Fig. 1:**
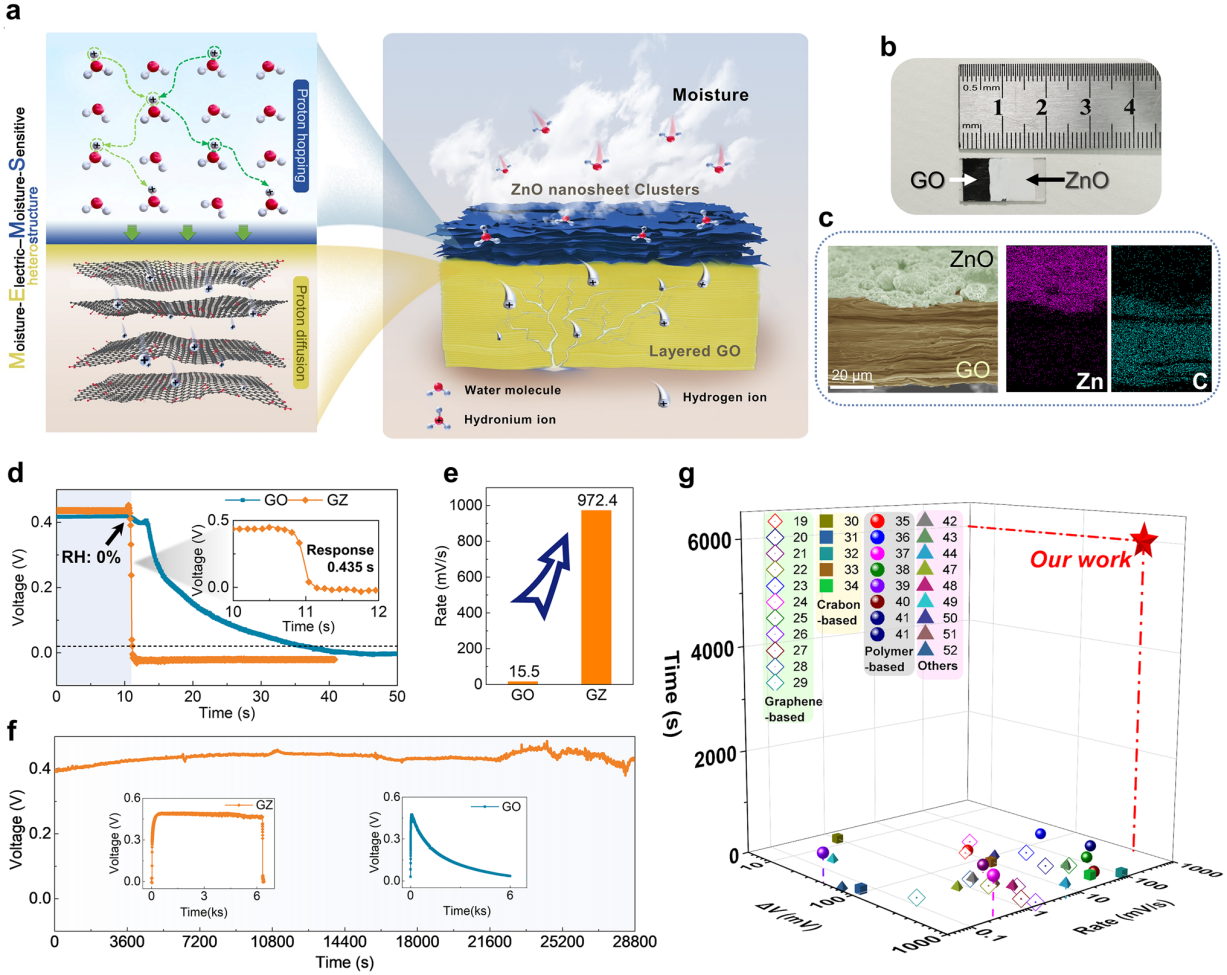
Design and characterization of GZMEG. **a** Design of GZMEG. GZMEG consists of ordered layered GO as ME material and loose ZnO nanosheet clusters as MS material, allowing moisture entry/escape. Proton hopping inside ZnO is triggered by moisture, which accelerates the proton diffusion rate in GO. Benefiting from the well-designed structure, GZMEG can provide ultra-fast response and efficient power output under alternating moisture stimulation. **b** Photograph of GZMEG. **c** SEM image of GO-ZnO heterostructure cross-section and element mapping images of zinc and carbon, showing clear delamination of the GO-ZnO heterostructure. **d** Voltage response of GO MEG (GOMEG) and GZMEG to moisture removal. Inset in (**d**) shows the entire ultrafast response of the GZMEG to dry N_2_ in 0.435 s. **e** Voltage average response rates of GOMEG and GZMEG for moisture removal. **f** A continuous voltage output of GZMEG for 8 h in the ambient environment (~ 90% RH, 25 ± 5 °C). The insets show the GZMEG with the voltage reaching its maximum value and continuing to output until the moisture is removed, and the GOMEG that the voltage gradually drops to zero over 6,000 s. **g** Systematic comparison of continuous voltage output and voltage responses based on reported various materials. Unless otherwise specified, moisture at 90% RH was used for all humidification processes, and dry N_2_ was used for all dehumidification processes

Herein, a graphene oxide (GO)-zinc oxide (ZnO) ME generator (GZMEG) was developed by constructing a moisture-electric GO and moisture-sensitive ZnO heterostructure, which utilizes the fast hopping of Grotthuss protons in the moisture-sensitive ZnO to regulate the heterostructure interface potential and obtains the ability to have an ultra-fast response to moisture and a long-time sustained and stable electrical output (Fig. 1a). Compared with the previous MEG by harnessing a single proton diffusion process, the proton hopping significantly reduces the diffusion barrier for proton migration in ZnO and GO, allowing a built-in interfacial potential at the interface of the GO-ZnO heterostructure, which can effectively improve the response rate of MEG and prolong the voltage output. The GZMEG achieves quick open-circuit voltage response (0.435 s) to alternating moisture stimuli, a record 972.4 mV s^−1^ response rate and stable output for over 8 h. Benefiting from the excellent sensitivity and stability of GZMEG, a high-precision respiratory monitoring alarm device and obstructive sleep apnea hypoventilation syndrome (OSAHS) diagnostic system of GZMEG was developed to monitor hypoventilation and apnea in humans in real-time and to successfully perform early warning diagnosis. This work contributes to the development of efficient moisture energy harvesting and demonstrates the great potential of ME-MS heterostructured MEGs derived from interfacial potential alignment for personal health monitoring and small smart medical electronics, paving the way for the preparation of novel MEG.

## Experimental Section

### Materials

Flake graphite (325 mesh, purity > 99.5%) was provided by XFNANO Technology Co., Ltd (Nanjing, China). Sodium nitrate (NaNO_3_, AR), sulfuric acid (H_2_SO_4_, AR), potassium permanganate (KMnO_4_, AR) and hydrogen peroxide (H_2_O_2_, 30%wt) were bought from Xilong Scientific Technology Co., Ltd (Shantou, China). ZnO (99.9%, 200 nm) was supplied by Meryer Chemical Technology Co., Ltd (Shanghai, China). Al_2_O_3_ (99.9%, 200 nm), Fe_3_O_4_ (99.5%, 100–300 nm) and TiO_2_ (99.8%, 100–300 nm) were purchased from Aladdin Biochemical Technology Co., Ltd (Shanghai, China). ITO conductive glass (0.7 mm thickness, 1 × 2 cm^2^, square resistance: 7 ohms), FTO conductive glass (1.1 mm thickness, 1 × 2 cm^2^, square resistance: 7 ohms) and soda-lime glass (1 mm thickness) used in the preparation of gold.

### Preparation of Electrodes

Typically, ITO conductive glass was used as the electrode of GZMEG. The ITO conductive glass was washed three times alternately with ethanol and deionized water, dried in an oven (60 °C) for 12 h, and then used to etch patterned electrodes. A 1 × 2 cm^2^ ITO conductive glass was etched with a laser (5 W) along the length to form a 5 mm wide non-conductive area (Fig. S10a). In Fig. S23, gold electrodes, and FTO conductive glass electrodes were prepared in the same way. The soda-lime glass (1 × 2 cm^2^) is used as the substrate of gold electrodes, and a layer of gold film is plated on it as the etch patterned electrode (current: 10 mA, sputtering time: 30 min) with Ion sputtering instrument (SD-3000, Boyuan Micro Nano Co., Ltd.). The FTO conductive glass (1 × 2 cm^2^) was washed three times alternately with ethanol and deionized water, dried in an oven (60 °C) for 12 h, and then used to etch patterned electrodes. Uniform holes were manufactured on the soda-lime glass for moisture passage to prepare microporous gold electrodes, and the holes’ diameter was 1 mm (Fig. S1b). The customized porous glass is used as the substrate, and a layer of gold film is plated on it as the electrode (current: 10 mA, sputtering time: 30 min) with an Ion sputtering instrument (Fig. S1c). Microporous gold electrodes and ITO conductive glass were applied in tests for graphene oxide ME generator (GOMEG) and ITO conductive glass served as the bottom electrode of GOMEG. The conductive carbon paste electrodes were obtained by coating conductive carbon paste on a 1 × 2 cm^2^ glass sheet having the same size as the ITO electrodes.

### Preparation of GZMEG

GO dispersion (7 mg mL^−1^) was synthesized by oxidation of natural graphite powder using the modified Hummers’ method reported previously [27]. The ZnO nanoparticles were prepared into a 20 wt% aqueous dispersion, followed by magnetic stirring for 2 h to uniformly disperse. GO dispersion was screen printed on the patterned ITO conductive glass electrode and then dried in a constant temperature oven of 35 °C for 12 h to form a 1 × 1 cm^2^ GO film. Subsequently, the ZnO aqueous dispersion was screen printed on the top of GO film and dried in a constant temperature oven of 35 °C for 2 h to obtain a 1 × 1 cm^2^ ZnO layer. The relative position of the GO film and the ZnO layer is shown in Fig. S10a. Similarly, MEGs based on other moisture-sensitive oxides (Al_2_O_3_, Fe_3_O_4,_ and TiO_2_) were prepared in the same manner.

### Preparation of Graphene Oxide ME Generator

The GO dispersion (7 mg mL^−1^) was directly screen printed on non-laser-etched ITO conductive glass and dried in a constant temperature oven of 35 °C for 12 h to form a 1 × 1 cm^2^ GO film. A microporous gold electrode was placed on top of the GO film and fixed with a tape-sealed clip, resulting in a sandwich-structured GOMEG (Fig. S3).

### Material Characterization

The morphology and microstructures of samples were characterized by a scanning electron microscope (SEM, SUPRA 55, Zeiss) with an energy dispersive spectroscopic (EDS). Transmission electron microscopy (TEM) and High-resolution TEM (HRTEM) images were carried out using field emission transmission electron microscopy (Talos F200X G2, FEI). The optical photographs were recorded on a Camera (Canon EOS 80D). X-ray diffraction (XRD) patterns were recorded on a Bruker D8 Advance X-ray powder diffractometer with a Cu Kα irradiation source (λ = 1.54 Å). X-ray photoelectron spectroscopy (XPS) was performed on an ESCA Lab 220i-XL electron spectrometer from VG Scientific using 300 W Al Ka radiation, and the spectrum were calibrated with the C 1*s* peak at 284.6 eV as an internal standard. Raman spectrum were obtained using an RM 2000 Microscopic Confocal Raman Spectrometer (Renishaw PLC, England) with a 532 nm laser. Kelvin probe force microscope (KPFM) images were taken using an Innova (Bruker) atomic force microscope. The zeta potential of moisture-sensitive oxide aqueous solution (10 μM) was analyzed by a Zeta potential analyzer (Malvern Zetasizer Nano ZS90) at pH = 7. The Fourier Transform Infrared spectrum (FT-IR) was performed on Thermo Scientific Nicolet iS20 in the 4000–400 cm^−1^ frequency range. Electrochemical impedance spectroscopy (EIS) was conducted on an AutoLab PGSTAT204 electrochemical workstation. The water contact angle was measured by a Lauda OSA contact angle goniometer. All voltage and current signals were acquired by a Keithley 2612 multimeter, which was controlled by a LabView-based data acquisition system. More details of the tests are shown in Note S1.

## Results and Discussion

### Fabrication and Electrical Output Performance of GZMEG

GZMEG is constructed by sequentially screen-printing GO and ZnO layers on laser-etched ITO conductive glass, where the ZnO layer covers half of the GO film area to ensure effective contact between GO and moisture while building a GO-ZnO heterostructure, as shown in Fig. 1b. The XRD pattern, SEM images, FTIR spectrum, Raman spectrum and XPS spectrum of GO confirm the synthesis of amorphous GO containing rich oxygen-containing functional groups (Figs. S14–S19, Note S4). The GO film is uniformly covered by stacked ZnO nanosheet clusters, which facilitate moisture entry into the ZnO interior, as shown in Fig. S5. The GO-ZnO heterostructure is confirmed to consist of ordered stacked GO films and loose ZnO nanosheet cluster layers with respective thicknesses of 40 and 7 μm, coinciding with the asymmetric distribution of zinc and carbon (Fig. 1c). The TEM image of the GO-ZnO heterostructure shown in Fig. S6a, indicates that the ZnO nanosheet cluster and the GO monolayer are tightly connected. Moreover, representative HRTEM images of the GO-ZnO heterostructure and corresponding FFT patterns of selected areas are given in Fig. S6b, demonstrating amorphous GO sheets and well-crystalline ZnO. The lattice spacing in HRTEM is measured to be 0.248 nm, corresponding to the (101) crystal plane of ZnO, while the FFT patterns are consistent with the XRD data (Fig. S7).

The GZMEG has electrodes on both sides connected directly to a precision meter placed in the relative humidity (RH) control system shown in Fig. S10b. Unless otherwise specified, all tests alternate between 90% RH and dry N_2_ to produce an alternating moisture and dry test environment. For comparison with the output electrical signals of the GZMEG, a GO ME generator (GOMEG) was also constructed, where the GO was sandwiched between a pair of gold electrodes (Fig. S3a, Note S2). During successive blowing of moisture into the GOMEG and GZMEG, the voltages gradually increased to a stable value decreased after blowing dry N_2_ (Fig. S12a). For the voltage rise from 0 V to the same value (0.3 V), it was observed that the GZMEG took only 1.895 s, in contrast to GOMEG which took ~ 30 s (Fig. S12b, c). The average response rate of the GZMEG voltage was calculated to be as high as 153.43 mV s^−1^, which is 16 times faster than the 9.59 mV s^−1^ response rate of the GOMEG (Fig. S12c). Notably, the response time of GZMEG to dry N_2_ was as short as 0.435 s, while the voltage drop response time of GOMEG during the same level of dehumidification was 25.68 s (Fig. 1d). The average response rate of GZMEG even reached 972.4 mV s^−1^ (Fig. 1e), substantially exceeding the values reported for other MEGs under the same conditions summarized in Table S1. More importantly, GZMEG also holds long-time stable voltage output in addition to having a fast response to alternating humidity. The GZMEG can continuously output voltage for 8 h without attenuation in high humidity (90% RH) environments (Fig. 1f). According to previous research [19–22], the output voltage gradually decreases after reaching its maximum value, even if the high humidity environment is maintained, regardless of the form of GOMEG. In the insets of Fig. 1f, the voltage of GOMEG drops to 0 after 6,000 s, but the GZMEG maintains a high voltage output with ultrafast response under the same ambient conditions (~ 90% RH, 25 ± 5 °C). A systematic comparison of voltage value, response rate, and sustained output time for all reported MEGs is shown in Fig. 1g [19–44, 47–52]. Most conventional MEGs are pulsed voltages, and a few MEGs can last for a short time and then drop slowly. Only the GZMEG constructed from ME-MS heterostructures has an ultra-fast response to moisture and a long-time sustained and stable electrical output.

**Fig. 2:**
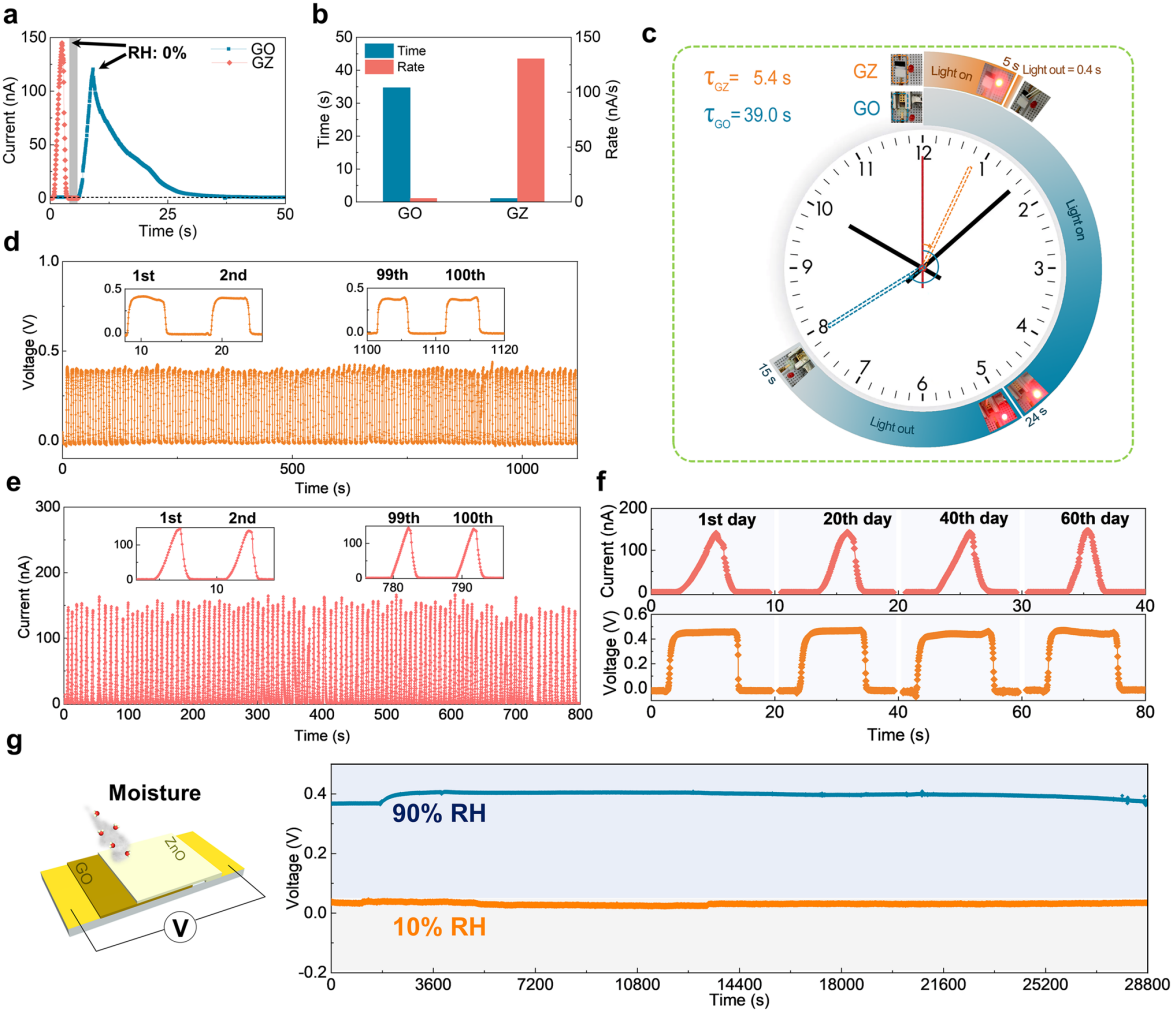
Electrical output performance of GZMEG. **a** Current response of GOMEG and GZMEG to moisture removal. **b** Current response times and average response rates of GOMEG and GZMEG for moisture removal. **c** Comparison of the response rates of GZMEG and GOMEG-controlled light-emitting diodes (LEDs) to moisture (~ 90% RH) and dry N_2_. Voltage (**d**) and current (**e**) stability of ultrafast response (100 cycles) for GZMEG. The insets show the first two cycles and the last two cycles, respectively. **f** GZMEG has stable ultrafast voltage and current responses on days 1, 20, 40 and 60 (ambient condition, ~ 40% RH, 25 ± 5 °C). **g** Continuous voltage output of GZMEG for 8 h at 90% RH and 10% RH after 60 days of shelving, demonstrating good robustness. Unless otherwise noted, moisture at 90% RH was used for all humidification processes, and dry N_2_ was used for all dehumidification processes

In addition, GO-ZnO heterostructure as an electricity-generating layer effectively shortened the current response time of dehumidification to within 1.1 s compared with GO, and the average current response speed of GZMEG was 38 times faster than that of GOMEG (Fig. 2a, b). Figure 2c and Movie S2 show the GZMEG and GOMEG controlling the process of turning on and off a light-emitting diode (LED). Impressively, the GZMEG takes only 5 s to fully light the LED, which is almost one-fifth of the time of GOMEG (24 s). Meanwhile, the GZMEG turns off the LED in 0.4 s, which is 1 ~ 2 orders of magnitude shorter than GOMEG (15 s). The above results consistently confirm that the proton hopping phenomenon generated in the GO-ZnO heterostructure dramatically increases the sensitivity of the MEG to alternating humidity changes. Figure 2d, e shows that the GZMEG exhibits excellent ultra-fast response stability for voltage and current over 100 cycles. GZMEG was still characterized by stable fast response after being shelved in a conventional environment for 1, 20, 40, and 60 days, as shown in Fig. 2f. Moreover, GZMEG can stably output voltage for 8 h at 90% RH and 10% RH without any attenuation, representing its excellent robustness (Fig. 2g). These contrasts imply that the GZMEG has significant advantages as a switch for humidity control and monitoring human respiration in situations where high response sensitivity is required, such as some higher frequency humidity changes.

### Verification of the Electricity-generating Mechanism

**Fig. 3:**
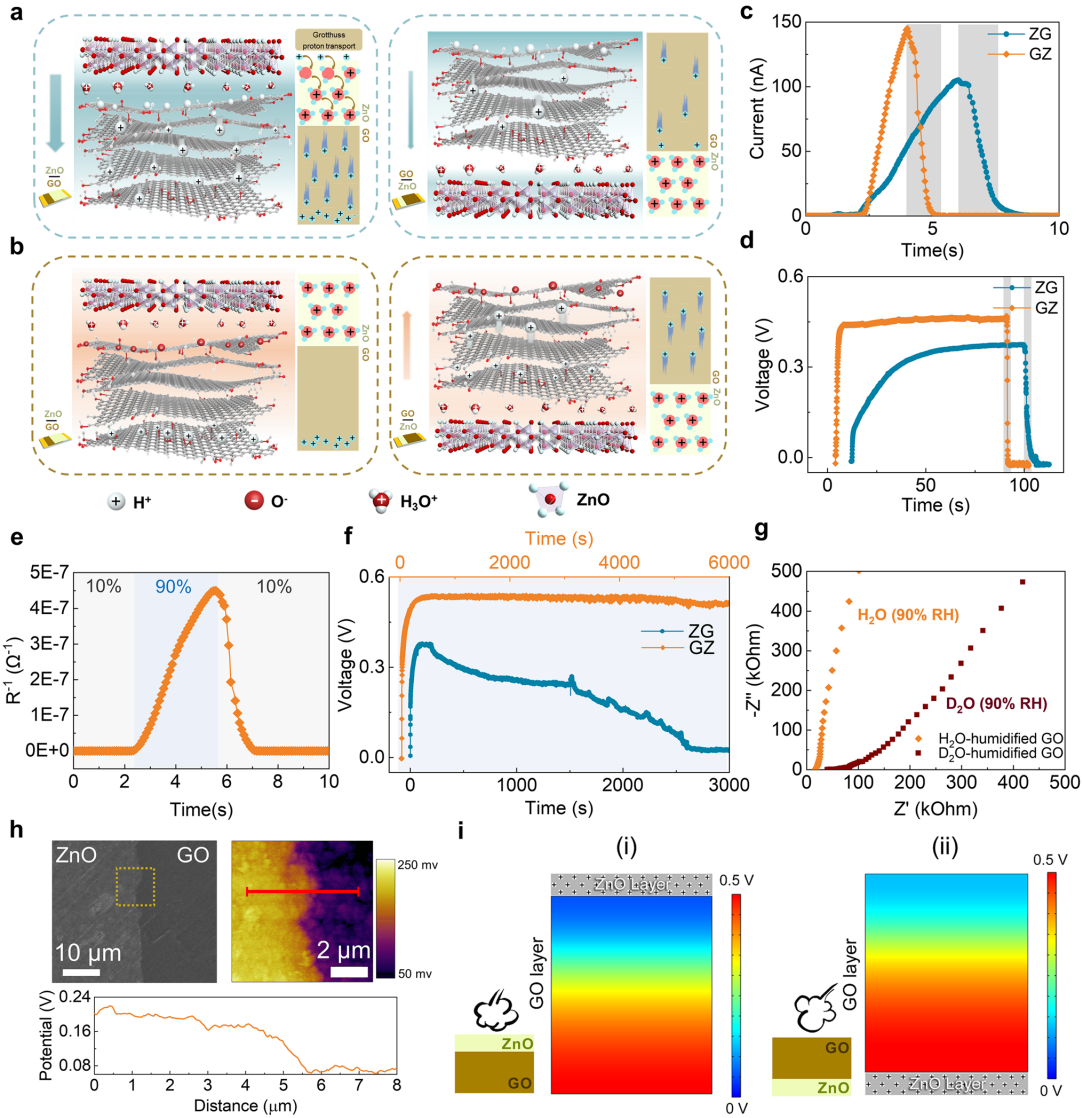
Mechanisms of GZMEG. **a** Schematic diagram of the current output mechanism of GZMEG and ZnO-GO MEG (ZGMEG), showing that the electropositive ZnO in the upper layer increases the current output and conversely limits the current output. **b** Mechanism of the continuous voltage output of GZMEG. The electropositive ZnO in the upper layer maintains a voltage balance and on the contrary breaks this balance. Current (**c**) and voltage (**d**) responses performance of GZMEG and ZGMEG. **e** Resistance changes of ZnO film under low (10%) and high (90%) RH. **f** Continuous voltage output performance of GZMEG and ZGMEG at 90% RH. **g** Nyquist plots of GO film in the presence of H_2_O and D_2_O at 90% RH. **h** Kelvin probe force microscopy images of GO-ZnO heterostructure at 90% RH. **i** Calculated the induced potential distribution of GO along the thickness direction when ZnO is in the upper (i) and lower layers (ii) of the heterostructure, respectively. Unless otherwise specified, moisture at 90% RH was used for all humidification processes, and dry N_2_ was used for all dehumidification processes

The conventional electricity generation of the moisture-electric GO is due to the spontaneous adsorption of water molecules through oxygen-containing functional groups to release free hydrogen ions, while the asymmetric moisture distribution drives the free diffusion and directed motion of carriers (Fig. S30). Therefore, the electrical response rate of the moisture-electric GO is slow, as reported in previous works [19–29]. We propose a Grotthuss proton hopping mechanism in a ME-MS GO-ZnO heterostructure to explain the ultrafast electrical response rate of GZMEG. When water molecules come in contact with the surface of the negative characteristic moisture-sensitive ZnO [63], the water molecules are ionized into H^+^ and OH^−^ by the exposed Zn^2+^ and O^2−^. Thus, the first layer of adsorption, i.e., chemisorption, is formed on the surface of ZnO, where Zn^2+^ are bound to OH^−^ and H^+^ are bound to O^2−^. As shown in Fig. S31, the other layers of water molecules formed physical adsorption with the previous layer of water molecules through single hydrogen bonds with increasing humidity. The greater local charge density and electrostatic field strength around the chemisorbed OH^−^ prompted the decomposition of water molecules in the physisorbed state to H_3_O^+^ under the action of the electrostatic field (H_2_O + H_2_O → H_3_O^+^ + OH^−^). H_3_O^+^ and then release H^+^ to the second water molecule, forming a Grotthuss proton transport (H_2_O + H_3_O^+^ → H_3_O^+^ + H_2_O) [64], which eventually generates a large amount of H_3_O^+^ in the continuous water film (Fig. S31). Similarly, the side of GO exposed to moisture is also flooded with a large amount of H_3_O^+^ [65]. When GZMEG is in a high-humidity environment, the Grotthuss mechanism greatly reduces the diffusion barrier for proton migration in ZnO and GO, allowing a built-in interfacial potential at the interface of the GO-ZnO heterostructure due to the flooding with a large amount of positively charged H_3_O^+^. The built-in interfacial potential accelerates the directional diffusion of the ionized H^+^ in GO, allowing the GZMEG to generate ultra-fast current and voltage responses, as shown in the left of Fig. 3a.

In addition, it is possible to consider moisture-sensitive ZnO as an external resistor in the GZMEG (Fig. S32a). At high humidity, the adsorption of water molecules on the surface of ZnO gradually increases, generating large amounts of H_3_O^+^. Hydrogen ions are transferred between H_3_O^+^ and water molecules through the Grotthuss mechanism to transport the charge, resulting in a sharp decrease in the resistance of ZnO to 10^6^ Ω (Fig. 3e). At this time, the small resistance of ZnO does not affect the electrical signal output (Fig. S3f), and thus the ultra-fast response rate of GZMEG to moisture can be observed. Blowing dry N_2_ into the ZnO surface abruptly increases its resistance by 4 orders of magnitude to 10^10^ Ω in an ultra-short time (Fig. 3e), at which point the ZnO is equivalent to an open circuit (Fig. S32b), and the electrical signal of the moisture-electric GO immediately disappears. Unlike the MEG composed of individual GO, the asymmetric potential distribution inside the GO layer in GZMEG does not return to the initial state after the end of dehumidification. During the subsequent humidification process, the electrical signal rapidly recovers, mainly because the moisture-sensitive ZnO resistance rapidly decreases, which enables the GZMEG to rapidly generate the electrical signal again. Unusually, the GZMEG can produce a relatively constant output voltage when placed in the same high RH environment, while the voltage of GOMEG fails to exhibit a long-term sustained output (Fig. 1f). This is because the internal electric field of GO drives the H^+^ to gradually return to its initial position and complex with the anionic groups, leading to the disappearance of the ion concentration gradient. In contrast, the built-in interfacial potential of the GO-ZnO heterostructure well suppresses the backward diffusion of hydrogen ions in GO (see left side of Fig. 3b), thus enabling the generation of a long-lasting and stable voltage. Conventional MEGs usually rely on the construction of asymmetric gradients of RH or functional groups that can dissociate carriers in a single ME component to achieve a stable output through free diffusion and directional migration of carriers. If the asymmetric gradient disappears, the electrical signal gradually decreases to the initial level.

Controlled experiments and numerical simulations were used to further validate the mechanism of built-in interfacial potential. The ZnO-GO heterostructured MEG (ZGMEG) was prepared by exchanging the ZnO layer with the GO layer. It can be found that the humidification electrical response rate of ZGMEG is much slower than that of GZMEG, while the dehumidification response time is similar, as shown in Fig. 3c, d. This is because the built-in potential formed by the ZnO-GO heterostructure in the lower part of the GO layer inhibits the directional diffusion of the H^+^ ionized in the GO (right of Fig. 3a), resulting in very slow voltage and current growth. The fast response of dehumidification also stems from the rapid increase of the system resistance, so the elapsed time for the disappearance of the electrical signal of the two constitutively opposite heterostructures is essentially the same. Moreover, Fig. 3f shows that the voltage of the ZGMEG is also unsustainable at high humidity, dropping to zero within more than 2,000 s after reaching its peak, even faster than the voltage disappearance of the GOMEG. This phenomenon is thought to be caused by the positive interfacial potential at the ZnO-GO heterostructure below the GO layer, which promotes H^+^ returns from the lower layer of GO to its original position of the upper layer and recombines with the anionic group, resulting in the disappearance of the electrical signal (as shown in the right of Fig. 3b).

To verify that proton hopping is governed by the Grotthuss mechanism, we further investigated the kinetic isotope effect (KIE) of GO film and the ratio of the film conductivity in H_2_O vapor and D_2_O vapor. The conductivity was calculated as follows:

1 $$\sigma =\frac{L}{R\bullet S}$$

where σ is conductivity, *L* is the length of film, *S* is the cross-sectional area of film, and *R* is the resistance calculated from Nyquist plots (Fig. 3g). For the same sample humidified with H_2_O and D_2_O,

2 $$\frac{{\sigma }_{H}}{{\sigma }_{D}}=\frac{{R}_{D}}{{R}_{H}}$$

where σ_H_ is the conductivity of H_2_O-humidified GO film, σ_D_ is the conductivity of D_2_O-humidified GO film, *R*_H_ is the resistance of the H_2_O-humidified GO film, and *R*_D_ is the resistance of the D_2_O-humidified GO film. For the Grotthuss mechanism, the KIE is usually no less than (*m*D/*m*H)^1/2^ ~ 1.4 [66–68]. The conductivity of H_2_O-humidified GO film is 2.3 times higher than that of D_2_O-humidified GO film, confirming that proton hopping is controlled by the Grotthuss mechanism. In addition, the Nyquist plot for the D_2_O-humidified GO film shows a distinct Warburg region accounting for sluggish ion transport [69]. In contrast, the Warburg region of the H_2_O-humidified GO film is much shorter, representing better ion diffusion [70].

In Fig. 3h, a drop of GO aqueous dispersion with a concentration of 0.2 mg mL^−1^ was placed on the prepared ZnO nanosheet, and the potential distribution at the ZnO-GO heterostructure was observed by Kelvin probe force microscopy (KPFM). It can be seen that there is a clear potential dividing line at the ZnO-GO heterostructure at 90% RH, with the potential on the ZnO side being higher than that on the GO side. The cross-sectional plot of the potential distribution shows that the potential of ZnO is about 120 mV more elevated than that of GO, which implies that more positive carriers are generated on the ZnO surface at high humidity. The calculated results show that the potential of GO is distributed along the thickness direction when ZnO is located in the upper and lower layers of the heterostructure as shown in Fig. 3i. The simulated output voltages of GZMEG and ZGMEG are 0.45 V and 0.38 V, consistent with the experimental results, indicating the plausibility of the mechanism.

**Fig. 4:**
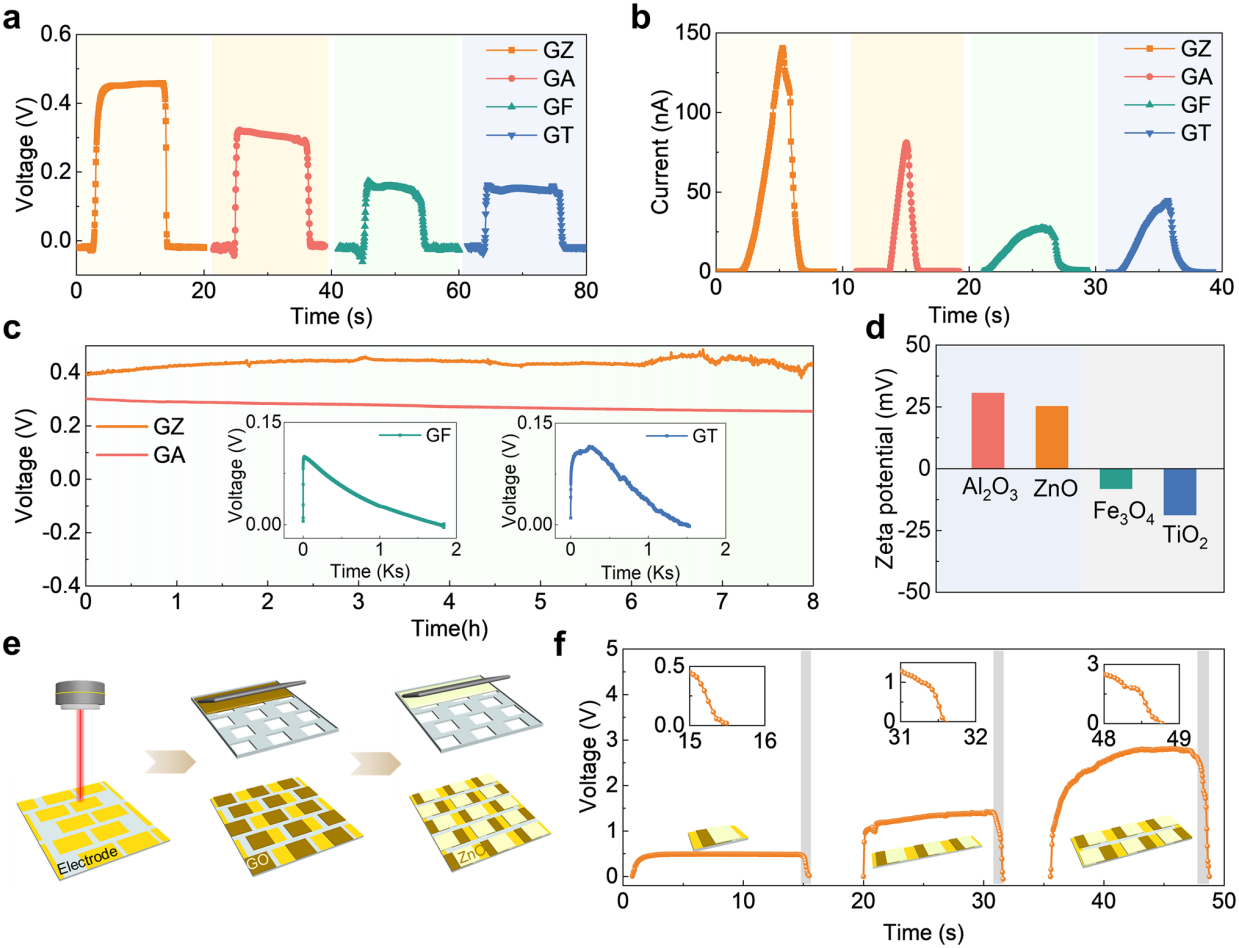
Performance of other GO-humidity sensitive oxides MEG and integrated GZMEG. Voltage responses (**a**) and current responses (**b**) of GZMEG, GAMEG, GFMEG, and GTMEG. **c** Continuous voltage output performance of GZMEG, GAMEG, GFMEG, and GTMEG in the ambient environment (~ 90% RH, 25 ± 5 °C). **d** Zeta potential (*ζ*) of Al_2_O_3_, ZnO, Fe_3_O_4_, and TiO_2_ aqueous solution (10 μM, pH∼7.0). **e** Schematic illustration of the integrated GZMEG units by step-by-step screen printing. **f** Voltage responses of the integrated device with 1, 3, and 6 GZMEG units in series

The effect of zeta potential on Grotthuss proton hopping was next focused on. Several moisture-sensitive semiconductors with different zeta potentials were selected to construct ME-MS heterostructures with GO to investigate their electrical generation behaviors and ultrafast response mechanisms. GO-Al_2_O_3_ MEG (GAMEG) was built from the moisture-sensitive semiconductor Al_2_O_3_ with positive zeta potential, while GO-Fe_3_O_4_ MEG (GFMEG) and GO-TiO_2_ MEG (GTMEG) were constructed from the moisture-sensitive semiconductors Fe_3_O_4_ and TiO_2_ with negative zeta potential, respectively, and their response rates to moisture and long-term power output capability were tested, as shown in Fig. 4. The GAMEG with the same zeta potential characteristic Al_2_O_3_ involved in the composition has the same electrical characteristics as the GZMEG (Fig. 4a, b). The difference in maximum current and voltage is attributed to the difference in the intrinsic resistance of the moisture-sensitive semiconductors (Fig. S35, Note S5). However, the electrical response rate of the humidification process is much slower for GFMEG and GTMEG, especially for the currents. Notably, the GAMEG and GZMEG were also able to maintain a stable voltage output for up to 8 h, while the voltage of the GFMEG and GTMEG gradually dropped to zero within 2,000 s (Fig. 4c). We believe that the opposite built-in interfacial potentials at the ME-MS heterostructures are the essential reason why GFMEG and GTMEG exhibit electrical characteristics different from those of GZMEG. The zeta potential test in Fig. 4d shows that the surfaces of ZnO and Al_2_O_3_ are positively charged, while the surfaces of Fe_3_O_4_ and TiO_2_ are negatively charged. The presence of the ME-MS heterostructures means that the interface where Fe_3_O_4_ and TiO_2_ are tightly bound to GO is filled with negative charges, which greatly inhibits the downward diffusion of H^+^ in the GO layer during the humidification process and severely delays the electrical response time (Fig. S36a). On the other hand, the built-in interfacial negative potential promoted the redistribution of H^+^ after the voltage output equilibrium, leading to a faster voltage drop in GFMEG and GTMEG (Fig. S36b). These results indicate the prevalence of the ME-MS heterostructure mechanism.

GZMEGs can be designed with electrode patterns and combined with stepwise screen-printing methods to increase the output voltage (Figs. 4e and S37). The voltage of the integrated device increases exponentially with the number of series-connected GZMEGs while maintaining an ultra-fast response rate, especially during dehumidification when the voltage can drop to zero in less than 1 s (Fig. 4f).

### Application of GZMEG as an HRM

**Fig. 5:**
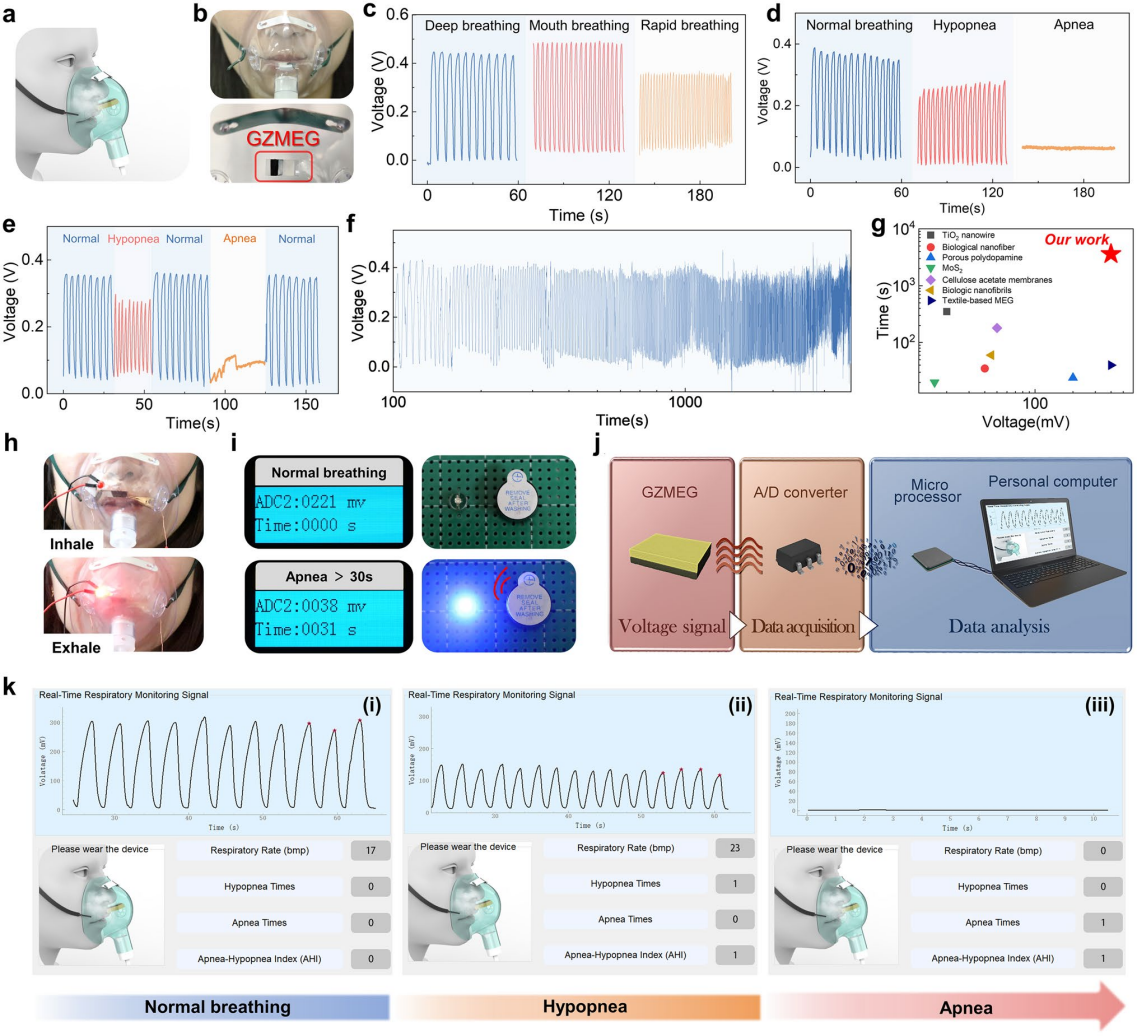
Demonstration of GZMEG as an HRM. **a** Schematic of a face mask with GZMEG as a HRM. **b** Photograph of an adult wearing a respiratory monitoring mask (top) and its internal structure (bottom). **c** Voltage signals of GZMEG under different respiratory modes. **d** Voltage signals of GZMEG under different respiratory states. **e** Real-time voltage signals of GZMEG during different respiratory state transitions. **f** Continuous respiratory monitoring using the GZMEG for 1 h. **g** Comparison of monitoring time and output voltage with reported MEG for respiratory monitoring. **h** Real-time responses of the GZMEG-controlled respiratory indicator to human respiration. The LED goes off during inhalation and comes on during exhalation. **i** Apnea alarm controlled by the GZMEG. Top: Alarm light and buzzer go off in a normal breathing state. Bottom: Alarm light on and buzzer alarm after a respiratory pause of more than 30 s. The experiments in (**h**) and (**i**) were completed on Jan. 16, 2022, in Beijing, China, with an ambient humidity of about 15% RH. **j** Schematic diagram of data acquisition and processing design of the GZMEG-based OSAHS diagnostic software. **k** GZMEG-based OSAHS diagnostic system for respiratory status determination. The experiment was completed on Apr. 22, 2022, in Beijing, China, with an ambient humidity of about 30% RH

The sensitivity of the GZMEG to rapidly alternating humidity gives it a huge advantage in monitoring human respiration quickly and with high accuracy. The GZMEG is designed as a Human Respiration Monitor (HRM) as shown in the schematic diagram in Fig. 5a and the physical images in Fig. 5b. The GZMEG is fixed in a suitable position in the mask to facilitate the sensing of human respiration. The intensity, rate, and status of the volunteer's breathing were monitored by recording the voltage response voltage of the GZMEG (Fig. S38). The real-time recording of different respiratory patterns including deep breathing, mouth breathing, and fast breathing showed that the GZMEG-based respiratory monitor could accurately distinguish different breathing states (Fig. 5c). Among them, the fast breathing was 40 times min^−1^, corresponding to the breathing frequency of infants and children, indicating that the respiratory monitor is suitable for people of all ages. Besides, the respiration monitor can accurately measure the intensity of normal breathing, hypopnea, and apnea, which are the criteria for obstructive sleep apnea–hypopnea syndrome (Fig. 5d). The very smooth and responsive real-time monitoring signal for different respiratory state transitions means that the GZMEG-based respiratory monitor can stably monitor complex and variable respiratory states (Fig. 5e). Based on the above characteristics of the GZMEG, the respiratory status of volunteers was monitored in real-time for up to 1 h and it was found that each respiratory cycle could be well recorded without significant fluctuations (Fig. 5f). The output voltage and monitorable duration of the GZMEG are much higher than other reported MEGs with similar features [47–52], as shown in Fig. 5g and Table S2.

If breathing is suspended for 30 s or more during sleep, it can lead to organ damage or even death and other dangers. The GZMEG-controlled indicator can monitor human breathing in real-time, and its design schematic is shown in Fig. S39a (Note S6). When the volunteer inhales, the mask is filled with dry air from the external environment, and the LED goes off. During exhalation, the GZMEG responds quickly to the exhaled moisture and lights up the LED (Fig. 5h). The GZMEG was tested as an HRM module in a real scenario (Fig. S39b). As shown in Fig. 5i, the alarm is turned off under normal breathing, and the output voltage of GZMEG varies with each breath. Once apnea begins and the output voltage of GZMEG falls below the alarm threshold, the alarm will start timing. After the apnea time exceeds 30 s, the alarm light will turn on and the buzzer alarm wakes the patient or transmits it to the monitoring personnel, thus avoiding sudden death. Real-time monitoring of respiration and apnea by the GEMEG-controlled respiration indicator light and alarm are demonstrated in Movies S3 and S4, respectively. The GZMEG-based respiration monitor was further developed to monitor and prevent OSAHS under conditions that significantly improve the response of the MEG to moisture. OSAHS is a common breathing disorder in humans during sleep, and its symptoms include apnea and hypopnea during sleep, which can cause sleep snoring, unresponsiveness, dizziness, and headache [71]. Long-term breathing disorders can also lead to hypoxemia, hypertension, and cerebral infarction, which can have a great impact on the health of patients. Polysomnography (PSG) is the current standard method for diagnosing OSAHS, but its procedure is cumbersome, bulky, and unsuitable for real-time sleep monitoring. Figure 5j shows the OSAHS diagnostic system, including the GZMEG module, data acquisition module, real-time data analysis, and interface output platform. Hypopnea is defined as a decrease in the strength (amplitude) of respiratory airflow during sleep of more than 30% from baseline level and lasting for 10 s, while apnea is defined as a complete cessation of oral and nasal respiratory airflow for more than 10 s [72]. After a period of measurement, the OSAHS diagnostic system using the GZMEG module displays the real-time respiratory signal and respiratory rate during the test and can accurately distinguish between normal breathing, hypopnea, and apnea (Fig. 5k). Besides, the Apnea–hypopnea index (AHI) is a criterion for determining the severity of OSAHS, defined as the average number of apnea and hypopnea per hour of sleep [73]. The system can also calculate the AHI during sleep, which determines the presence and severity of obstructive sleep apnea. The long-term monitoring of human respiration and OSAHS diagnosis by the GZMEG-based diagnostic system are shown in Fig. S40 and Movie S5, which successfully monitored the respiratory status within 1 h in real-time and recorded the number of apnea and hypopnea, thereby obtaining the current AHI (23.82), representing moderate OSAHS.

## Conclusions

A MEG with ultra-fast response to moisture and long-time sustained and stable electrical output capability was developed using a constructed ME-MS GO-ZnO heterostructure. The built-in interfacial potential of the GO-ZnO heterostructure, formed by the Grotthuss proton hopping in the moisture-sensitive ZnO, allows GZMEG to achieve quick response (0.435 s) and a record ultra-fast electrical response rate (972.4 mV s^−1^) while fundamentally solving the problem of the gradual decay of the output voltage of conventional ME materials. Based on this, we have developed a GZMEG-based apnea alarm and OSAHS diagnostic system that can distinguish between normal breathing, hypopnea and apnea during breathing, record the number of breathing disorders and diagnose OSAHS. The preparation of a new class of “moisture-electric” + “moisture-sensitive” heterostructured materials has facilitated the development of new key technologies for ME and given scientists a deeper understanding of the mechanisms of moisture-generated carrier migration in ME materials. This breakthrough discovery in the field of ME could provide a promising candidate for portable, fast-response human intelligent devices.

## Supplementary Information

Below is the link to the electronic supplementary material.

Supplementary file1 (MP4 1739 kb)

Supplementary file2 (MP4 4987 kb)

Supplementary file3 (MP4 2847 kb)

Supplementary file4 (MP4 8259 kb)

Supplementary file5 (MP4 16042 kb)

Supplementary file6 (PDF 2475 kb)
