# Supplementary material for: Moisture-Electric–Moisture-Sensitive Heterostructure Triggered Proton Hopping for Quality-Enhancing Moist-Electric Generator
Source: Nanomicro Lett. 2023 Dec 18;16:56. doi: 10.1007/s40820-023-01260-w (PMC10728039; doi:10.1007/s40820-023-01260-w)
Supplement: Supplementary file 6 — Supplementary file6 (PDF 2475 kb) [file 40820_2023_1260_MOESM6_ESM.pdf]

Supporting Information for

Moisture-Electric-Moisture-Sensitive Heterostructure Triggered Proton Hopping for Quality-Enhancing Moist-Electric Generator

Ya'nan Yang[1,2], Jiaqi Wang[1], Zhe Wang[1], Changxiang Shao[1], Yuyang Han[1], Ying Wang[1], Xiaoting Liu[1,2], Xiaotong Sun[1,2], Liru Wang[1,2], Yuanyuan Li[1,2], Qiang Guo[1,2], Wenpeng Wu[1,2], Nan Chen[1,2,]*, Liangti Qu[3]

[1] Key Laboratory of Cluster Science, Ministry of Education of China, Key Laboratory of Photoelectronic/Electrophotonic Conversion Materials, School of Chemistry and Chemical Engineering, Beijing Institute of Technology, Beijing 100081, P. R. China

[2] Yangtze Delta Region Academy of Beijing Institute of Technology, Jiaxing 314019, P. R. China

[3] Department of Chemistry, Key Laboratory of Organic Optoelectronics & Molecular Engineering, Ministry of Education, Tsinghua University, Beijing 100084, P. R. China

*Corresponding author. E-mail: gabechain@bit.edu.cn (Nan Chen)

Note S1 Tests and Methods

Construction of test equipment. The ME device consists of a Relative Humidity (RH) control system, a Keithley 2612 multimeter and a humidity sensor (Fig. S10b). MEG equipment is directly connected to the Keithley 2612 multimeter to test open-circuit current and short-circuit voltage. The circuit parameter of the open-circuit voltage test was current = 0 nA. The circuit parameter of the short- circuit current test was voltage = 0 mV. To avoid the influence of static electricity, all samples were short-circuited before testing.

Method for controlling the loading of H_2O molecules in the blown-in gas (RH). The blowing gas with different water vapor loadings is realized by the device shown in Fig. S10b, and water vapor is generated by passing dry N_2 into a gas scrubber containing pure water. Valve I is used to control the blown-in gas rate for a high-water load (90% RH) and valve II is used to control the blown-in gas rate for a high dry N_2 (0% RH). Valve I and valve II work together to accurately control the amount of water vapor load in the blown-in gas. The voltage and current responses of GZMEG under moisture carried by different gases were tested by changing the gas inlet to argon and air. Unless otherwise specified, moisture at 90% RH was used for all humidification processes in responsiveness tests, and dry N_2 was used for all dehumidification processes.

Construction and testing of GZMEG as an HRM. GZMEG is fixed in a common medical mask at a position facing the nostrils to facilitate the sensing of human respiration. The electrical signals generated by the GZMEG are transmitted to the Keithley 2612 multimeter through wires. The magnitude and frequency of the voltage correspond to different breathing intensity and rates. Similarly, by connecting the GZMEG mask to the port of the apnea alarm and obstructive sleep apnea hypoventilation syndrome (OSAHS) diagnostic system, the respiratory status can be monitored in real-time.

Electrochemical Impedance Spectroscopy (EIS) testing. EIS was acquired on an AutoLab PGSTAT204 electrochemical workstation. The frequency range was 0.1-10 kHz and the magnitude of the modulation signal was set to be 0 V to avoid possible interference by the voltage induced. The impedance of GOMEG humidified by D_2O and GOMEG humidified by H_2O was then tested at 90% RH.

Note S2 Characterization and Electrical Output Performance of Graphene Oxide (GO) Film

Obtained by the method of solvent evaporation induced self-assembly, the GO as the electricity- generating layer has good flexibility (Fig. S1). The microstructure and morphology of the surface and cross-section of the GO film were investigated by scanning electron microscopy (SEM), showing the smooth surface and ordered layered stacking structure of GO (Fig. S2). In addition, from the energy dispersive spectroscopy (EDS) spectrum (Fig. S2d), the weight ratio of oxygen element is as high as 38%, which means that the GO film contains abundant oxygen-containing functional groups.

The electrical output performance of the GO moisture-electric (ME) generator (GOMEG) was tested after placing a microporous gold electrode on the top layer of GO directly dried on ITO conductive glass (Fig. S3a). Moisture is carried by N_2 flowing through deionized water, and the RH of the test system is changed by adjusting the flow rate of N_2, as shown in Fig. S10b. A voltage of 0.4 V and a current of 120 nA were generated in GOMEG under 90% RH, while the electrical output gradually decreased under the stimulation of dry N_2 (Fig. S3c, d). According to our previous report, this phenomenon is caused by the moisture gradient brought by asymmetric moisture stimulation on both sides of GO [S1]. The stacking structure of layered GO hinders the diffusion process of water molecules, and the side close to the moisture absorbs more water vapor than the side farther away, resulting in the hysteresis of water transfer (Fig. S3b). In addition, the lower electrode, acting as a moisture-isolating substrate, further strengthens the asymmetry of water content inside GO, and the GO on the side of the moisture-isolating substrate cannot effectively contact moisture, while the side far from the moisture-isolating substrate can be sufficiently wetted. The upper layer of the GO film with excellent hydrophilicity is directly exposed to moisture, and oxygen-containing functional groups, such as carboxyl groups, interact with water molecules to ionize carboxylate and H^+ ions. The interaction of moisture and GO is hindered by the moisture-isolating substrate, so there are very few H^+ ions in the lower layer. Driven by the concentration difference, H^+ ions diffuse from the upper layer to the lower layer with water molecules, and the carboxylate groups are fixed on the carbon skeleton of GO and cannot move. H^+ ions and carboxylate recombine with the withdrawal of moisture, and the output voltage and current drop to the initial state. Therefore, the mobile restricted functional groups and the directionally mobile H^+ ions realize the self-generated electricity effect of the GO film. Moreover, the output voltage and current of GOMEG vary with external resistance, as shown in Fig. 3Se, f. When the external resistance is greater than 10^5 Ω, the output current decreases and drops to 0 at $R=10^8$ Ω. The output voltage drops when the external resistance exceeds 10^7 Ω and becomes 0 at $R=10^9$ Ω.

To further verify the principle that the moisture gradient causes GO to generate electricity, both sides of the GO film without the moisture-isolating substrate were exposed to moisture simultaneously (Fig. S4a). The output voltage of the symmetric moisture-stimulated GO film is almost zero (Fig. S4c) due to the same degree of hydration inside the GO resulting in no ion migration (Fig. S4b). Furthermore, the left-right asymmetric moisture stimulation on GO induced a lateral moisture gradient (Fig. S4d, e) and GO also generated a voltage of 0.4 V (Fig. S4f).

Note S3 Characterization and Electrical Output Performance of ZnO

ZnO is a crystal with a hexagonal wurtzite structure (Fig. S7), and the morphology is irregular nanosheets with a size of 200-500 nm (Fig. S8). Irregular ZnO nanosheets are stacked together to form many holes that facilitate the entry of water molecules. A ZnO layer of the same size as that on the graphene oxide-zinc oxide ME generator (GZMEG) was screen-printed between the two electrodes, and then the ME performance of the ZnO layer was tested (Fig. S9a). Both the voltage

and current outputs of the ZnO coating are 0, which indicates that ZnO has no ME characteristics (Fig. S9b, c).

Note S4 Characterization and Electrical Output Performance of GZMEG

The process of preparing GZMEG by step-by-step screen printing is shown in Fig. S10a. The RH control system that measures the ME output of MEG consists of external N_2 sources, a sealed deionized water bottle, a sealed constant RH bottle, a Keithley 2612 multimeter, and a RH detector. The moisture is carried by N_2 through deionized water to control the RH of the MEG surface. The Keithley multimeter connected to the electrodes of the MEG measures the electrical output, and the RH detector indicates the real-time RH.

SEM images of the GO-ZnO heterostructure interface show that the ZnO coating with loose pores is in good contact with the GO film (Fig. S5). The water contact angle of ZnO prepared by powder tablet press is even close to 0° (Fig. S11a), representing that ZnO is super-hydrophilic, so the ZnO coating does not hinder the entry of water into the GO film. Moreover, the water contact angle of GO film is 51° (Fig. S11b), indicating that it has good hygroscopicity and can generate electricity when interacting with water.

The response speed of GZMEG to RH changes is greatly improved compared with GOMEG, as shown in Figs. S12d, e. The instantaneous response rate of GZMEG in the humidification process can reach 0.5 V/s, and the response rate of the dehumidification process even reaches 2 V/s, while the total response rate of GOMEG is close to 0 V/s. In addition, the current response of GZMEG to moisture is twice as fast as GOMEG (Fig. S13a, b). It is worth noting that the response of GZEMG to dry N_2 has a super-strong improvement, and its instantaneous response speed is as high as 450 nA/s (Fig. S13c).

The X-Ray Diffraction (XRD) pattern, Fourier Transform Infrared (FT-IR) spectrum, and SEM images of GO in the initial state of GZMEG and after 100 cycles of ME testing are shown in Figs. S14-S16. The XRD confirms the representative peak at $2\theta = 11.58°$, indicating a larger interlayer spacing in GO (ca. 0.76 nm) than in pristine graphite (ca. 0.335 nm). In addition, the FTIR spectrum shows typical peaks of O-H bonding, C=O bonding, C=C bonding, C-OH bonding and C-O bonding. The characterization results before and after testing are consistent, demonstrating no chemical or structural changes. Figures S17-S19 show the EDS, Raman, and X-ray Photoelectron Spectroscopy (XPS) spectrum of GO in the initial state of GZMEG and after 100 ME test cycles. After the performance cycle test, no obvious change in the atomic ratio of C and O of the GO film is found (Fig. S17). Raman spectrum are a widely used method for characterizing graphene-based materials and the Raman spectrum of GO shows an apparent disorder D band at 1340 cm^{-1} and graphitic G band at 1600 cm^{-1} [S4]. The I_D/I_G ratios of the GO in the initial state and after the cycle test are 0.937 and 0.939, respectively, indicating no apparent structural change happens during the cycling test. Furthermore, the high-resolution C 1s spectrum (Fig. S19b) of GO revealed the presence of C–C bonding (~284.5 eV), C–O bonding (~286.8 eV), and C=O (~288.5 eV) [S1], and the spectrum after the test is basically unchanged, indicating that the oxygen-containing groups are still retained.

The SEM images (Fig. S20) of the ZnO layer display no significant change in the microscopic morphology before and after 100 cycles of ME testing, and the XRD pattern and FTIR spectrum are shown in the Figs. S21 and S22. There is no change in the characteristic peaks of ZnO with hexagonal wurtzite structure, implying that the ZnO layer was stable on GZMEG. The sharp peaks at 424 and 560 cm^{-1} in the FTIR spectrum are the characteristic peaks of Zn–O bonding. The absorption peaks at 3440 and 1630 cm^{-1} were attributed to the stretching and bending vibration absorption peaks of ZnO surface hydroxyl groups or bridged hydroxyl groups. In the atmosphere at room temperature, water is adsorbed on the surface of metal oxides, and in most cases the water eventually dissociates to form adsorbed

hydroxyl groups. The above results show that the ME effect did not change the chemical composition of GO and ZnO, which means that the GZMEG has long- term stable performance.

To verify that external factors such as electrodes would not affect the ultrafast response characteristics of GZMEG, we prepared GZMEGs with different electrodes and stimulated them by moisture carried by different atmospheres. The GZMEGs with gold, FTO conductive glass, and CH8 conductive carbon paste as electrodes, respectively, show similar rapid response capabilities (Fig. S23). Among them, the peak value of the electrical signal of GZMEG with CH8 conductive carbon paste as the electrodes is slightly smaller than other GZMEGs, because the carbon paste is not as smooth as other electrodes, and the contact with GZMEG is relatively poor, which will affect the output signal. On the other hand, changing the N_2 source to argon or air to adjust the RH, GZMEG also has a fast response performance (Fig. S24). The results indicate that external factors could not affect the characteristics of GZMEG.

The voltage and current responses of GZMEG under different RH conditions are shown in Figs. S25 and S26 respectively. All tests were performed by alternately stimulating GZMEG with moisture at different RH and dry N_2. Under 10%-40% RH, the voltage output is close to 0, because the resistance of ZnO is large under low RH, which limits the voltage output (Fig. S3f). As the RH further increases, the output voltage of GZMEG gradually increases and the response to the stimulation of alternating moisture becomes more rapid, since the Grotthuss mechanism more obviously promotes the directional diffusion of the ionized H^+ in GO, and the resistance of ZnO is smaller under high RH, which has little limiting effect on voltage. It is worth noting that the current has the same performance as the voltage under different RH, but the current is 0 below 50%RH owing to the output of the current signal is more easily restricted by the resistance of the external circuit (Fig. S3f).

The output performance of GZMEGs with different coverage areas was tested with other conditions being the same, where the ZnO layer covered 25%, 40%, 50%, 60% and 75% of the GO film area, as shown in (Fig. S27). The GZMEGs exhibited excellent rapid response to alternating stimulus of moisture (90% RH) and dry N_2, but the magnitude of voltage and current showed a trend of first increasing and then decreasing with the increase of coverage area. The smaller output signals of GZMEG with 25% and 40% coverage area were attributed to less ZnO in the upper layer, resulting in the Grotthuss proton hopping phenomenon not being strong enough to encourage the diffusion of abundant H^+. The difference in the signal peaks for GZMEGs with 60% and 75% coverage areas was attributed to the greater intrinsic resistance of more ZnO, which bounds the signal output.

Different sizes of GZMEGs were prepared and their voltage and current responses are shown in Fig. S28. The sizes of GO and ZnO layers in the GZMEGs prepared in this work are 1×1 cm^2, and the dimensions of the two functional layers in the GZMEG from the control experiments are 0.5, 2, and 3 cm^2, respectively. There is no significant difference in the voltage responsivity and magnitude of the different sizes of the GZMEGs, as the potential difference between the upper and lower surfaces of the GZMEGs is unchanged. However, the current magnitude of GZMEGs is proportional to the device size. As the size increases, the number of directionally migrating H^+ increases with it, and a larger current is generated. The current density is the same for all GZMEGs.

Note S5 Working Mechanism of GZMEG

A ZnO flake with a diameter of 13 mm was prepared by a powder tablet machine, and then a drop of GO aqueous dispersion (0.2 mg/mL) was dropped on it and then dried at 35 °C for Kelvin probe force microscopy (KPFM) test (Fig. S33a). The SEM images show that the ZnO flake has a very flat surface, and a thin layer of GO adheres to the ZnO (Fig. S33b, c). The relative surface potential

was measured at the junction of the GO-ZnO heterostructure (Fig. S33d), which is the red frame in Fig. S33a. The test results are shown in Fig. 3h.

With the overall structure unchanged, GO-Al_2O_3 ME generator (GAMEG), GO-Fe_3O_4 ME generator (GFMEG), and GO-TiO_2 ME generator (GTMEG) were prepared by replacing ZnO with Al_2O_3, Fe_3O_4, and TiO_2 to generalize "built-in interfacial potential coordination of heterostructures" theory to other moisture-electric-moisture-sensitive heterostructures. The electrical output properties of Al_2O_3, Fe_3O_4, and TiO_2 under 90% RH demonstrate that these oxides do not have ME characteristics (Fig. S34). Their resistance varied rapidly with RH to ensure fast MEG response. The difference in maximum current and voltage is attributed to the difference in the intrinsic resistance of the moisture-sensitive oxides (Fig. S35). The resistances of Al_2O_3, Fe_3O_4, and TiO_2 are 25, 6, and 5.4 MΩ at 90% RH, and 10^{10}, 10^9, and 6×10^8 Ω under 10% RH, respectively. The electrical signals of the moisture-electric-moisture-sensitive MEG is shown in Figs. S3e and S3f to decreases with the increase of the external series resistance, and the moisture-sensitive oxide in the moisture-electric- moisture-sensitive MEG is equivalent to a series external resistance. At the same high RH (90%), the resistance of Al_2O_3 is ten times that of ZnO, so the output voltage and current of GAMEG are lower.

Note S6 Application of GZMEG as an HRM

Using the GZMEG-based HRM, a series of devices for judging human respiration status and monitoring respiratory diseases in real-time were developed, including a respiration indicator light, apnea alarm, and obstructive sleep apnea-hypopnea syndrome (OSAHS) diagnostic system. A very portable and simple HRM consists of a GZMEG, a mask, and gold electrodes, as shown in Fig. S38. This simple respiration monitor can be used for various functions. For example, by connecting an amplifier and an LED, the GZMEG can be used as a respiratory indicator (Fig. S39a). The LED could respond in sync with the respiration to indicate the respiration status. The apnea alarm is composed of GZMEG, data acquisition, voltage comparison, LCD screen, LED, and buzzer (Fig. S39b). When the apnea time exceeds 30 seconds, the alarm indicator lights and the buzzer sounds. In addition, we also developed an OSAHS diagnostic system, which successfully monitored the respiratory status within one hour in real time (Fig. S40).

Supplementary Figures

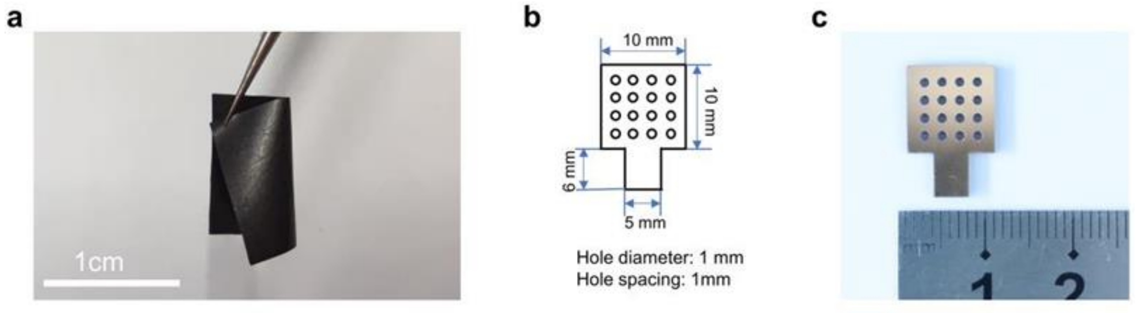

Fig. S1 a) Photograph of GO film. **b**) Dimensional diagram of the porous gold electrode. **c**) Photograph of a porous gold electrode

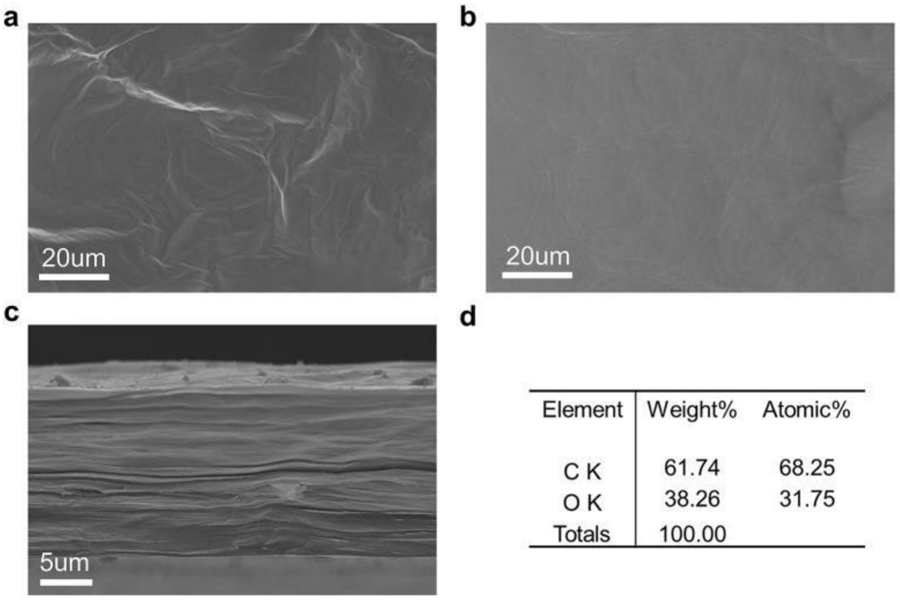

Element	Weight%	Atomic%
C K	61.74	68.25
O K	38.26	31.75
Totals	100.00	

Fig. S2 SEM images of GO film. **a**) Topside SEM view of GO. **b**) Bottom side SEM view of GO. **c**) Cross-sectional SEM view of GO. **d**) The EDS result of GO film

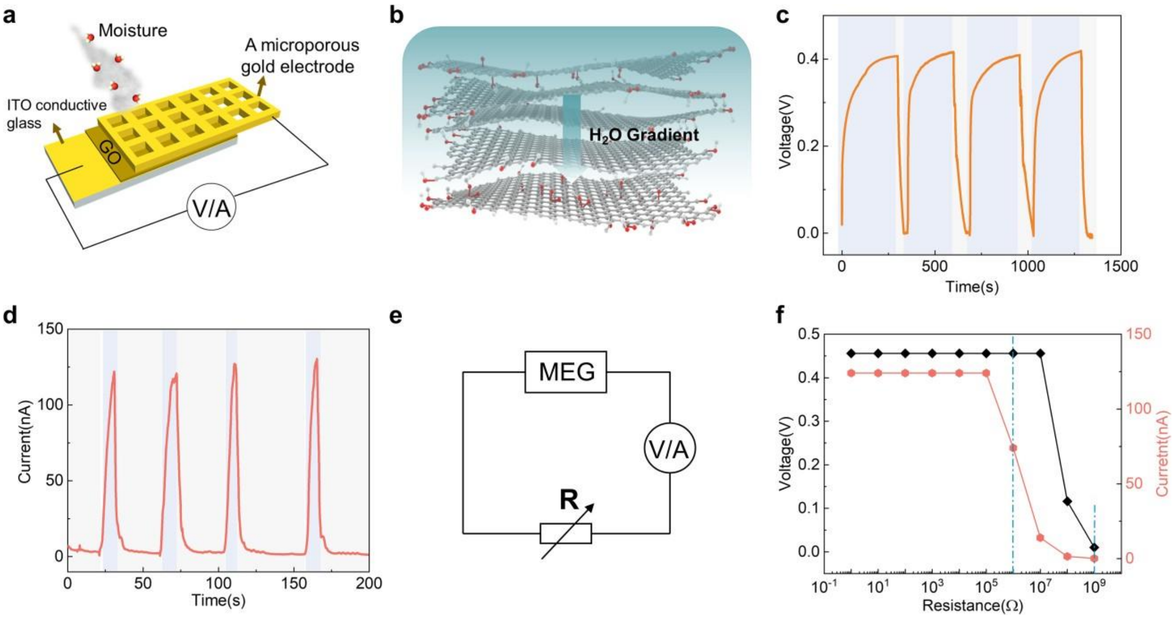

Fig. S3 Electrical output performance of GOMEG. **a**) Schematic diagram of testing the electrical output performance of GOMEG. **b**) Moisture gradient decreasing from top to bottom in GO film. **c**) Output voltage cycles under the alternating stimulation of moisture at 90% RH and dry N_2. **d**) Output current cycles under the alternating stimulation of moisture at 90% RH and dry N_2. **e**) Equivalent circuit diagram of the electrical output test of GOMEG with different external resistances. **f**) Output voltage and current of GOMEG with different external resistances at 90% RH, corresponding to (**e**)

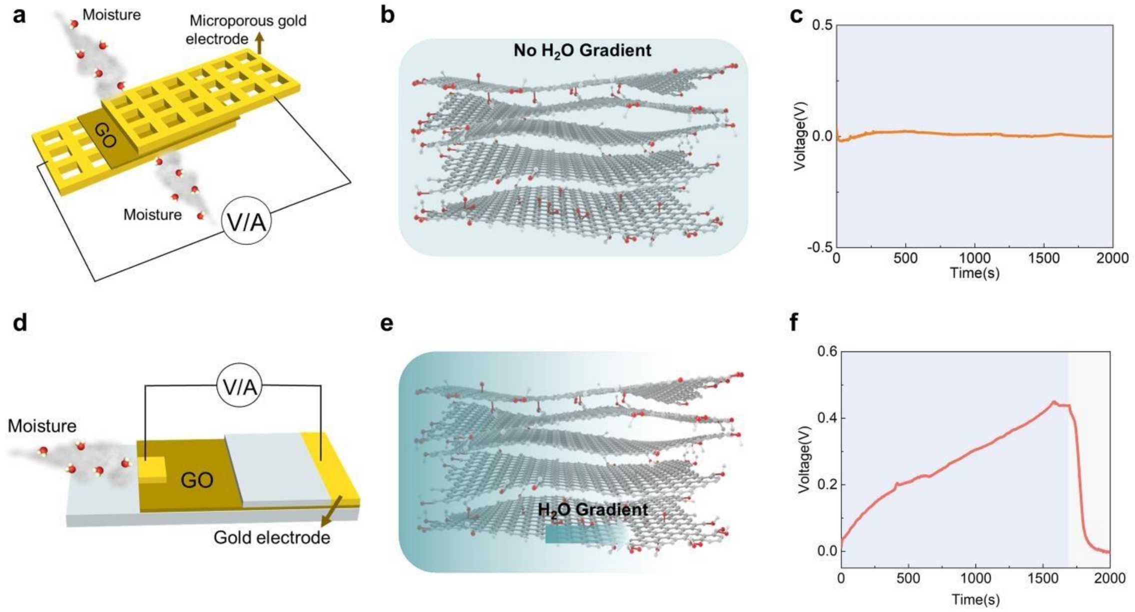

Fig. S4 Electrical output performance of GOMEG under other moisture stimulation methods. **a**) Schematic diagram of simultaneous moisture stimulation on the upper and lower sides of GOMEG. **b**) Without moisture gradient in simultaneously moisture-stimulated GO film. **c**) Output voltage of GOMEG at 90% RH according to (**a**). **d**) Schematic diagram of left-to-right asymmetric moisture stimulation to GOMEG. **e**) Moisture gradient decreasing from left to right in GO film. **f**) Output voltage of GOMEG at 90% RH according to (**d**)

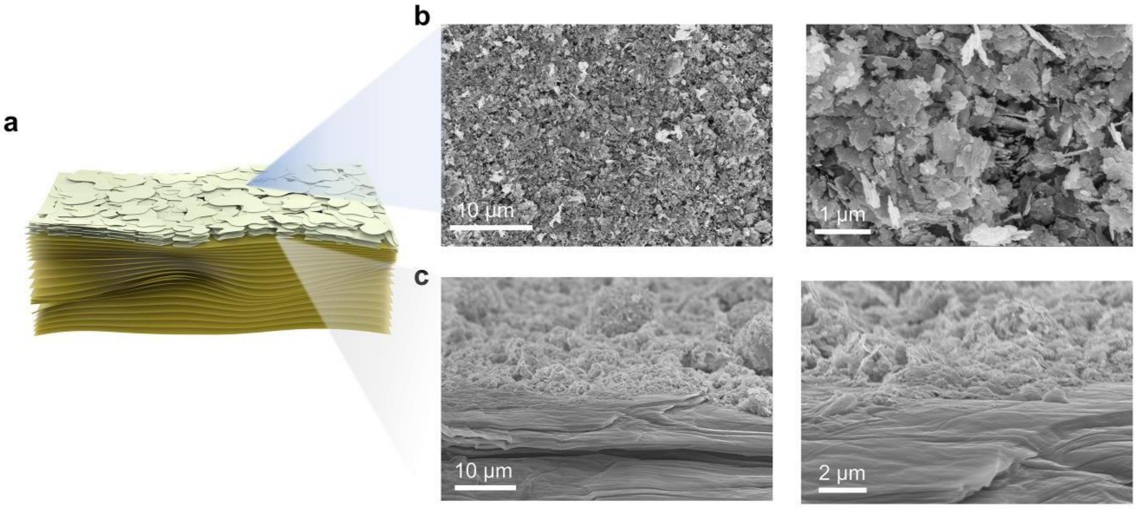

Fig. S5 a) Schematic diagram of GO-ZnO heterostructure. The upper layer is a ZnO layer, while the lower layer is an orderly stacked GO film. **b**) SEM images of the upper side of the GO-ZnO heterostructure at low and high magnification. **c**) SEM images of the GO-ZnO heterostructure interface

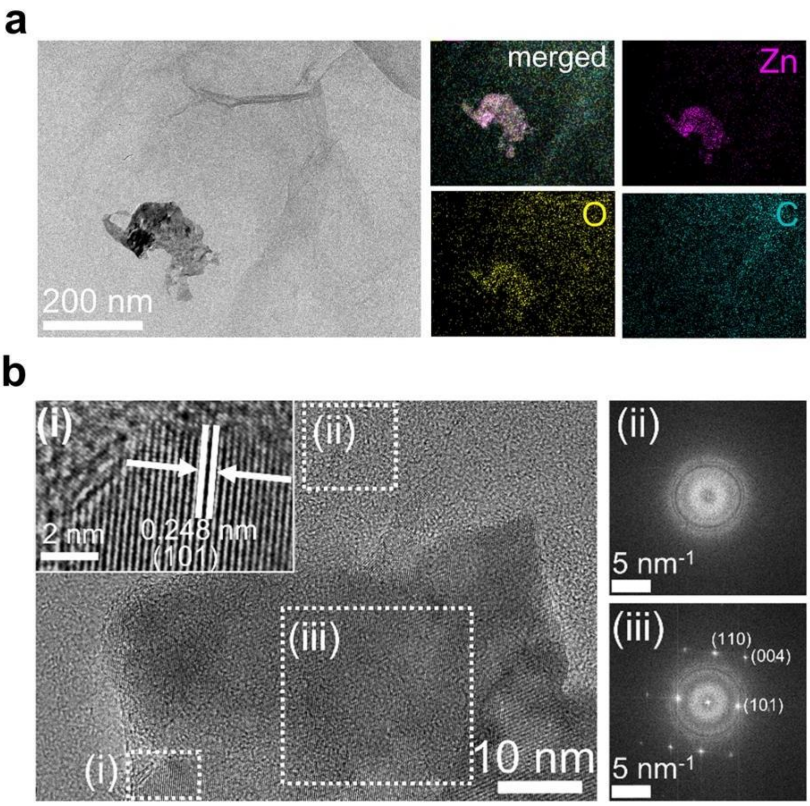

Fig. S6 Transmission electron microscopy (TEM) image of GO-ZnO heterostructure and the corresponding zinc, oxygen, and carbon mapping (**a**). High-resolution TEM (HRTEM) image of GO- ZnO heterostructure and the corresponding Fast Fourier Transformation (FFT) patterns of the selected areas (**b**)

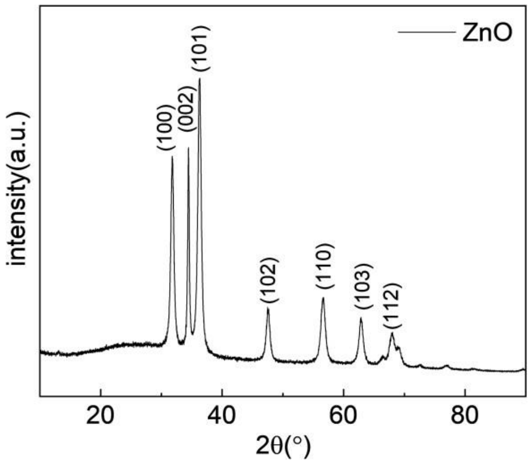

Fig. S7 XRD pattern of ZnO

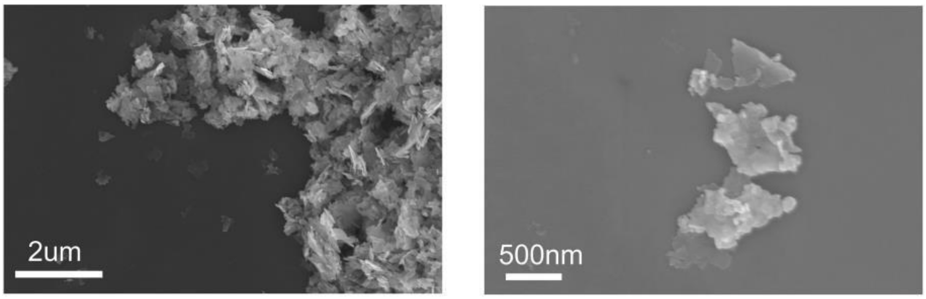

Fig. S8 SEM images of ZnO nanosheets

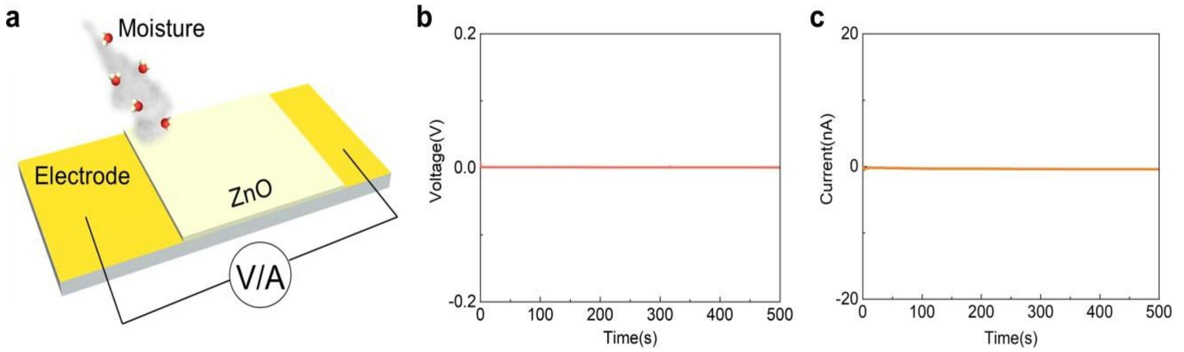

Fig. S9 Electrical output performance of ZnO. **a**) Schematic diagram of testing the electrical output performance of ZnO. Output voltage (**b**) and current (**c**) of ZnO under RH of 90%

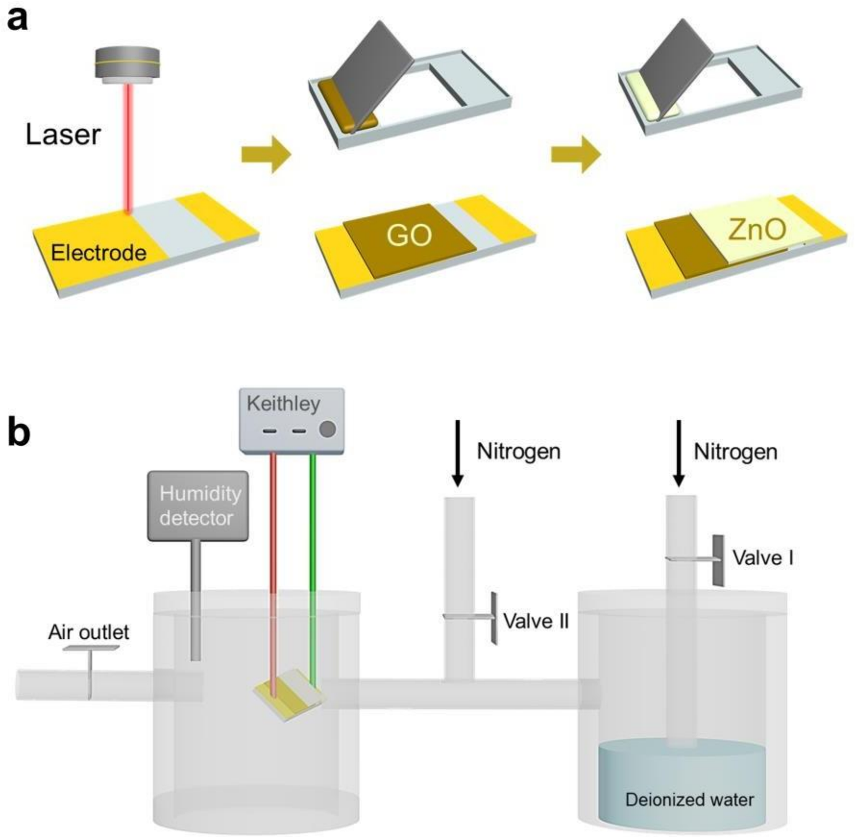

Fig. S10 Preparation process diagram (**a**) of GZMEG by a simple two-step screen-printing method and schematic diagram (**b**) of the RH control system for measuring the electrical output of MEG

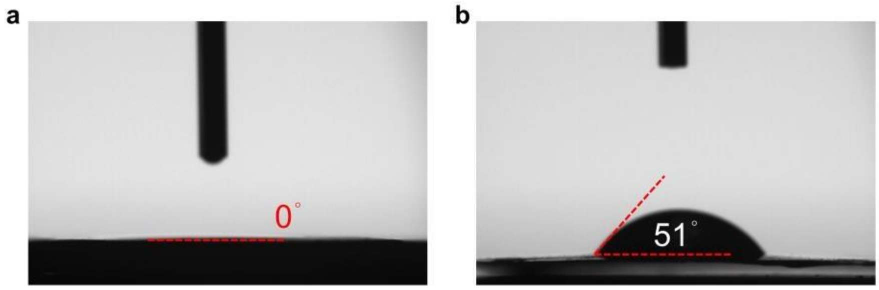

Fig. S11 Water contact angles of ZnO flake (**a**) and GO film (**b**)

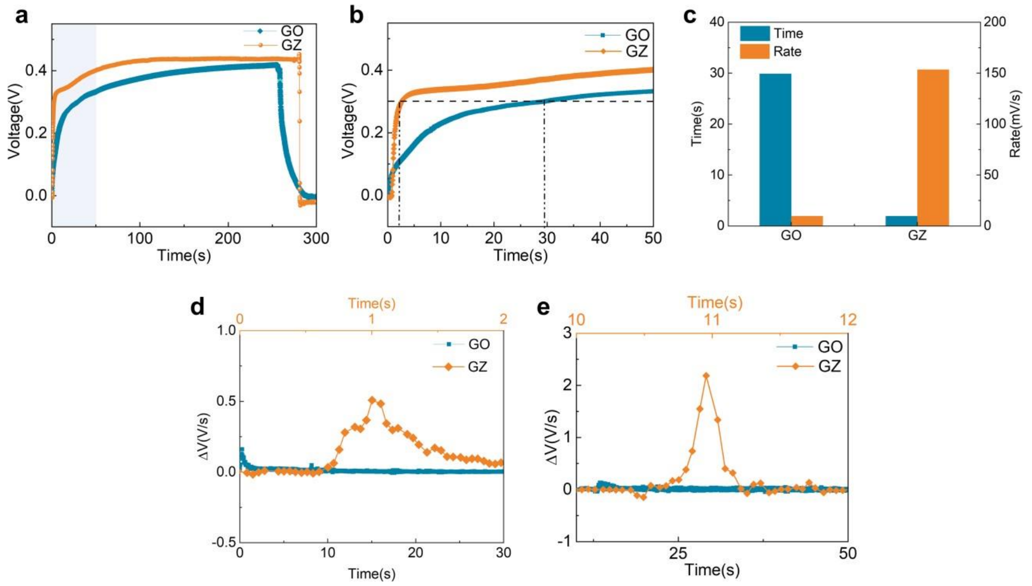

Fig. S12 Voltage responses of GZMEG. **a**) Voltage responses of GOMEG and GZMEG to humidification and dehumidification. **b**) Voltage responses of GOMEG and GZMEG to the humidification process. **c**) Voltage response time and average response rate of GOMEG and GZMEG to humidification process. The slope of voltage as a function of time for GOMEG and GZMEG during humidification (**d**) and dehumidification (**e**). Unless otherwise specified, moisture at 90% RH was used for all humidification processes, and dry N_2 was used for dehumidification processes

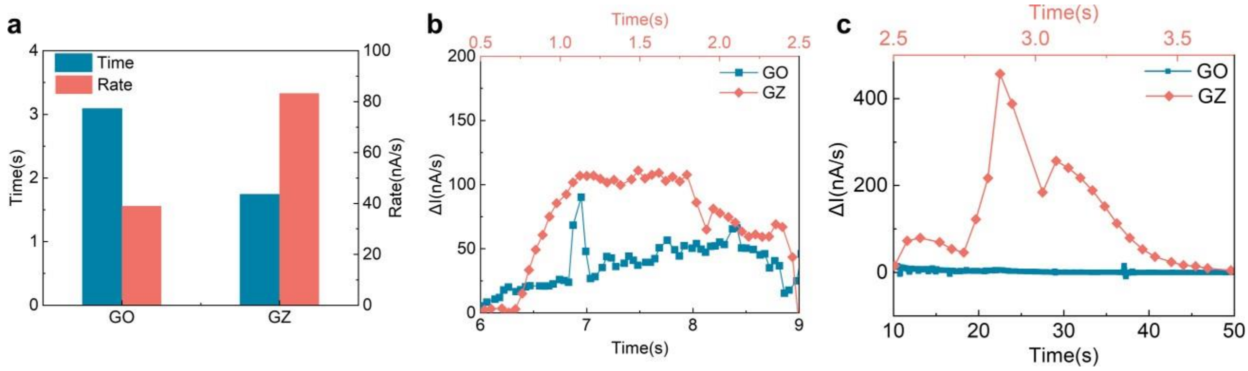

Fig. S13 Current responses of GZMEG. **a**) Current response time and average response rate of GOMEG and GZMEG to humidification process. The slope of current as a function of time for GOMEG and GZMEG during humidification (**b**) and dehumidification (**c**). Unless otherwise specified, moisture at 90% RH was used for all humidification processes, and dry N_2 was used for dehumidification processes

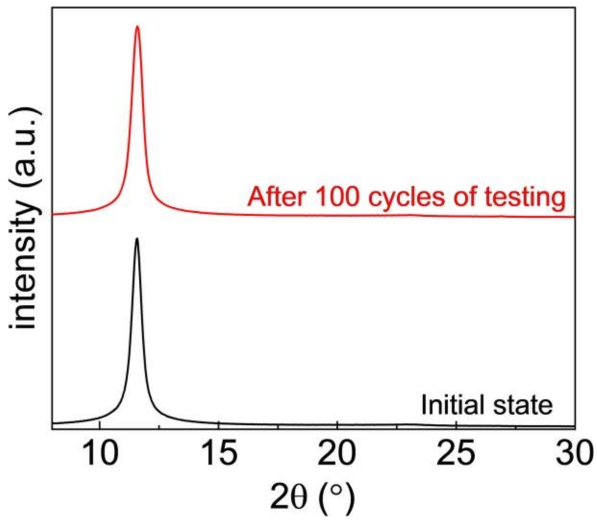

Fig. S14 XRD pattern of GO film in GZMEG at the initial state and after 100 cycles of ME testing

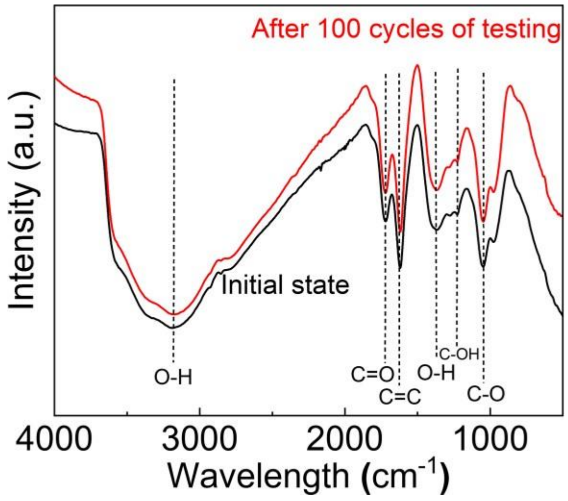

Fig. S15 FTIR spectrum of GO film in GZMEG at the initial state and after 100 cycles of ME testing

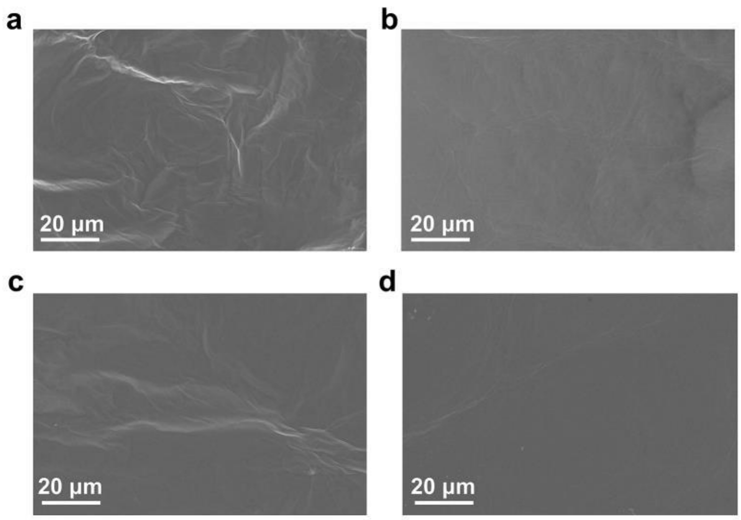

Fig. S16 SEM images of GO film in GZMEG at the initial state and after 100 cycles of testing. Top side (**a**) and bottom side (**b**) SEM view of GO at the initial state. Top side (**c**) and bottom side (**d**) SEM view of GO after 100 cycles of ME testing

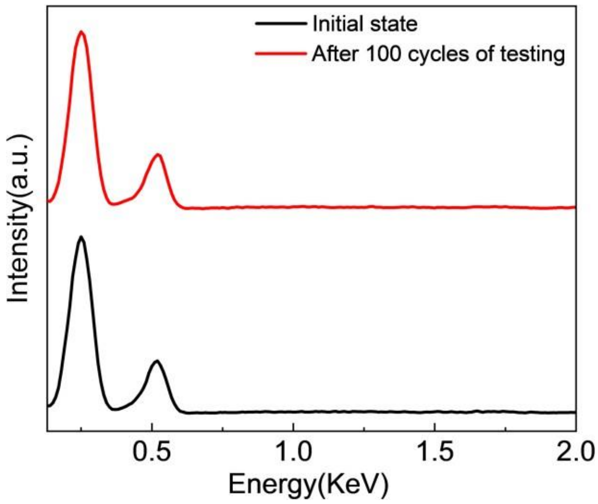

Fig. S17 EDS spectrum of GO film in GZMEG at the initial state and after 100 cycles of ME testing

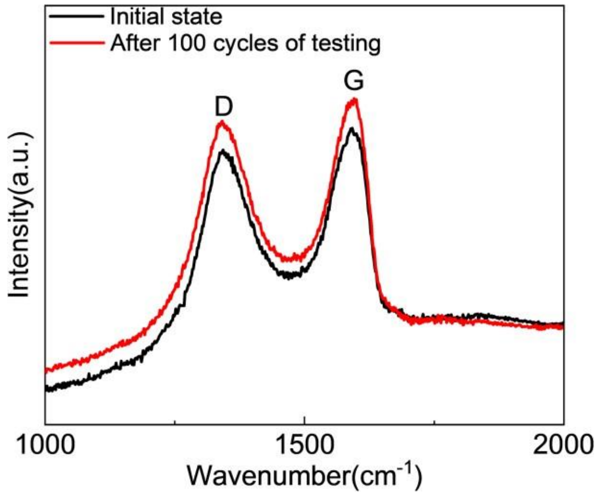

Fig. S18 Raman spectrum of GO film in GZMEG at the initial state and after 100 cycles of ME testing

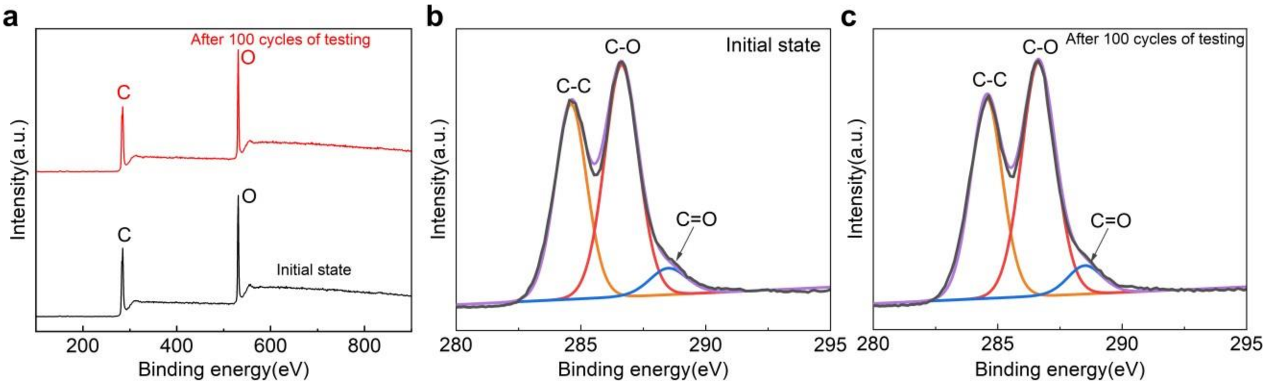

Fig. S19 XPS spectrum of GO film in GZMEG at the initial state and after 100 cycles of testing (**a**). The high-resolution XPS spectrum of C 1s peak of GO film at the initial state (**b**) and after 100 cycles of ME testing (**c**)

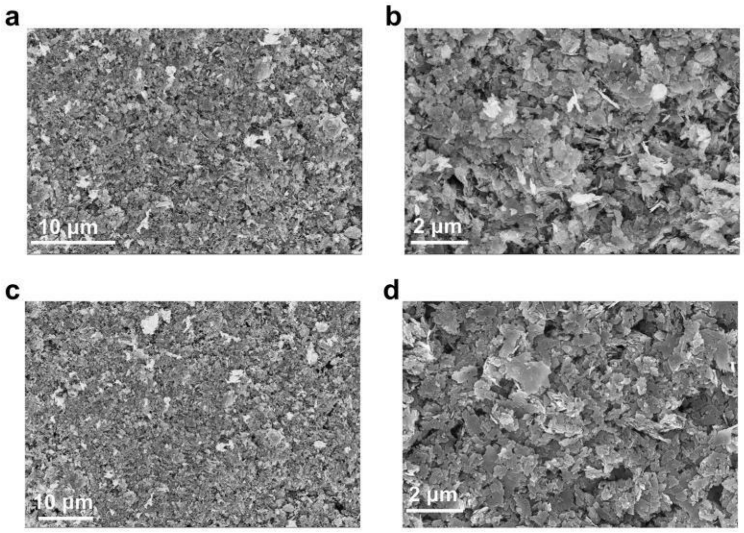

Fig. S20. SEM images of ZnO layer in GZMEG at the initial state and after 100 cycles of testing. (**a, b**) SEM view of ZnO at the initial state. (**c, d**) SEM view of ZnO after 100 cycles of ME testing

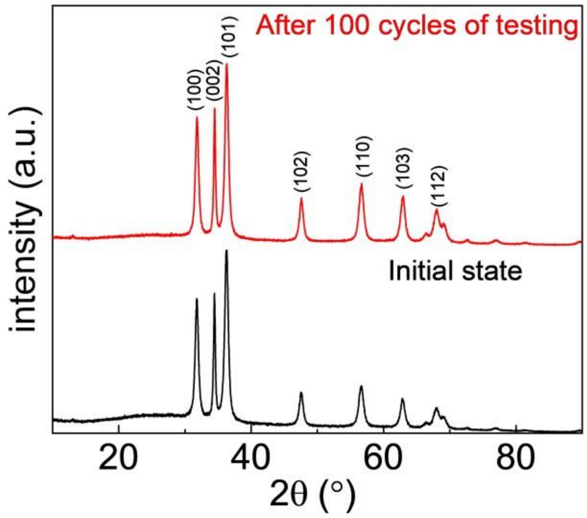

Fig. S21 XRD pattern of ZnO layer in GZMEG at the initial state and after 100 cycles of ME testing

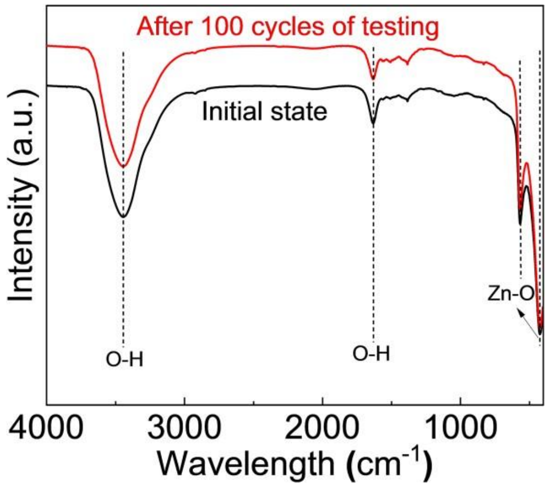

Fig. S22 FTIR spectrum of ZnO layer in GZMEG at the initial state and after 100 cycles of ME testing

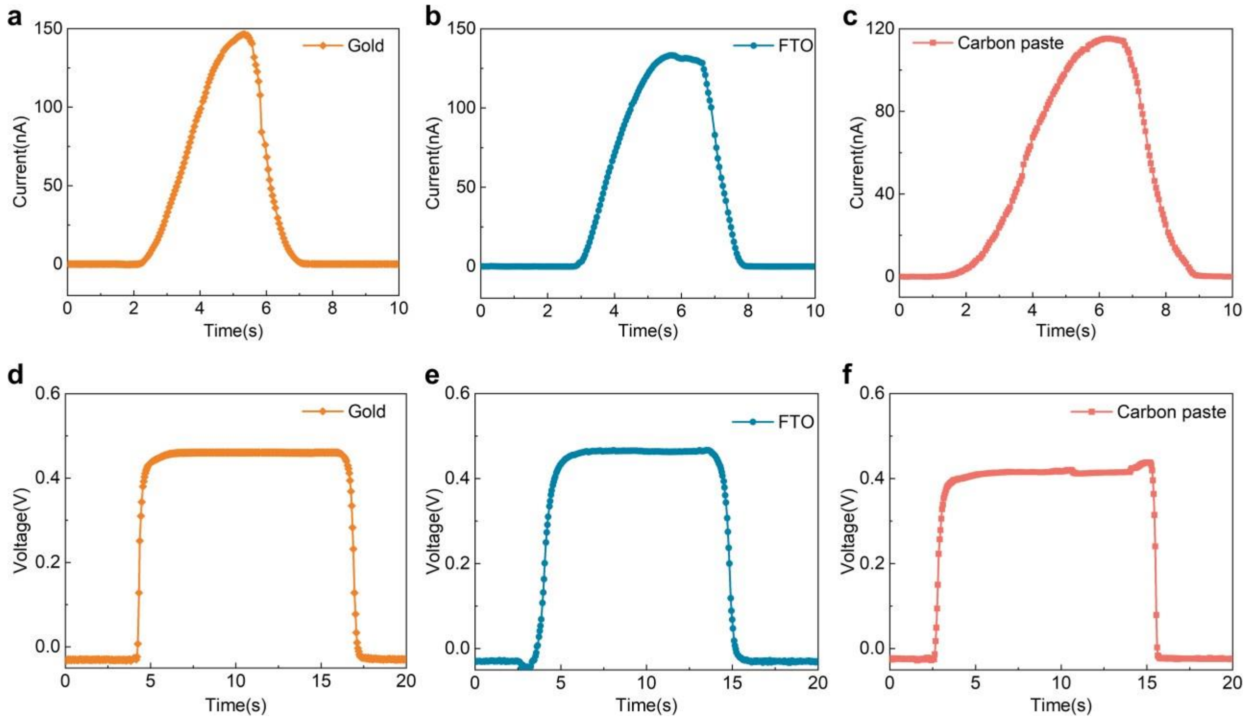

Fig. S23 Voltage and current responses of GZMEG with different electrodes. (**a, b**) Gold electrode. (**c, d**) FTO conductive glass electrode. (**e, f**) Conductive carbon paste electrode. Moisture at 90% RH was used for all humidification processes, and dry N_2 was used for dehumidification processes

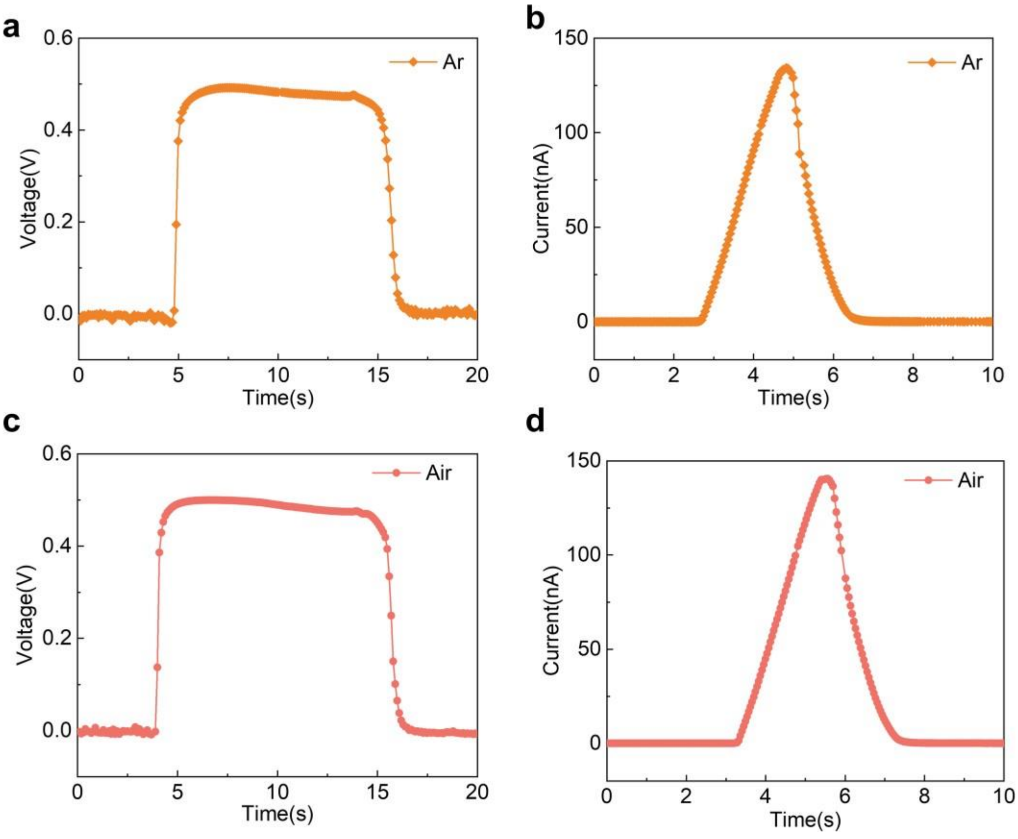

Fig. S24 Voltage and current responses of GZMEG under moisture carried by different gases. (**a, b**) Argon. (**c, d**) Air. Moisture at 90% RH was used for all humidification processes, and dry N_2 was used for dehumidification processes

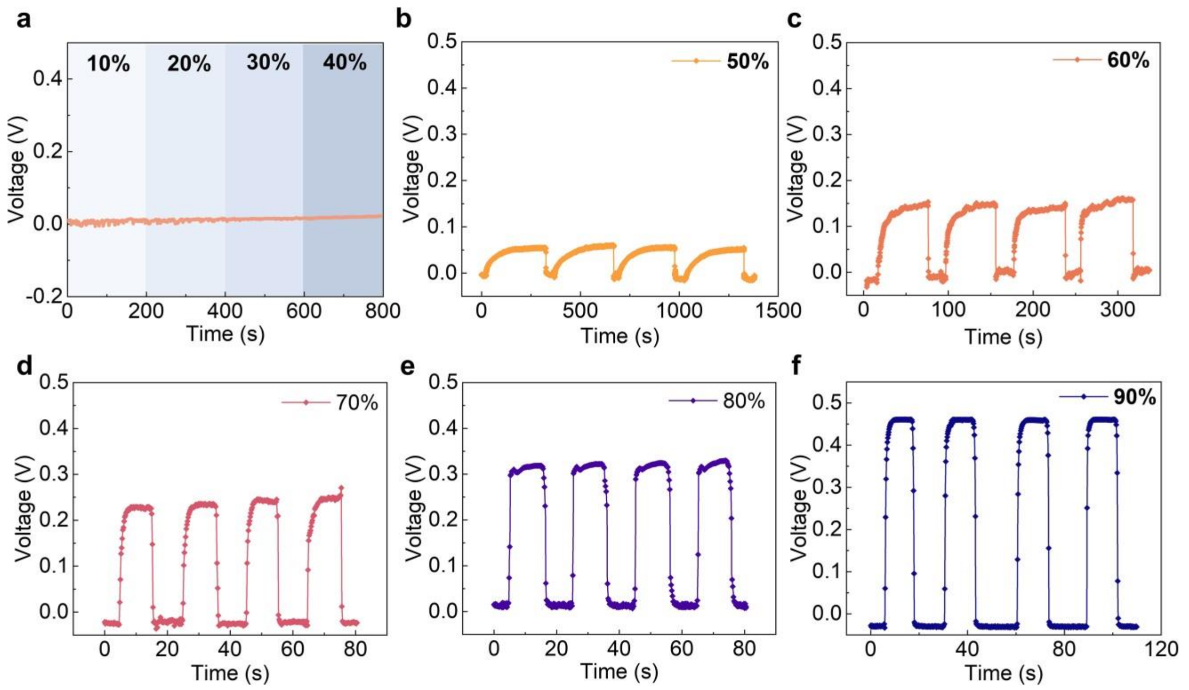

Fig. S25 Voltage responses of GZMEG under different RH. (**a**) 10%-40% RH. (**b**) 50% RH. (**c**) 60% RH. (**d**) 70% RH. (**e**) 80% RH. (**f**) 90% RH

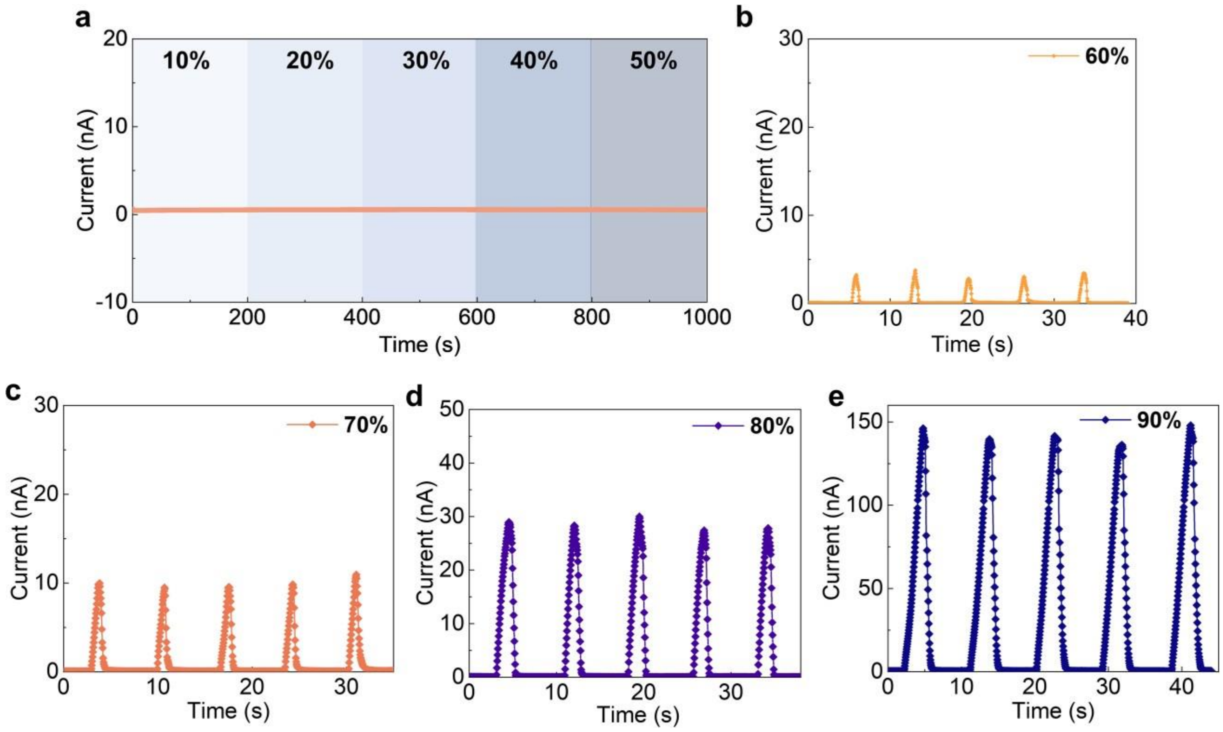

Fig. S26 Current responses of GZMEG under different RH. (**a**) 10%-50% RH. (**b**) 60% RH. (**c**) 70% RH. (**d**) 80% RH. (**e**) 90% RH

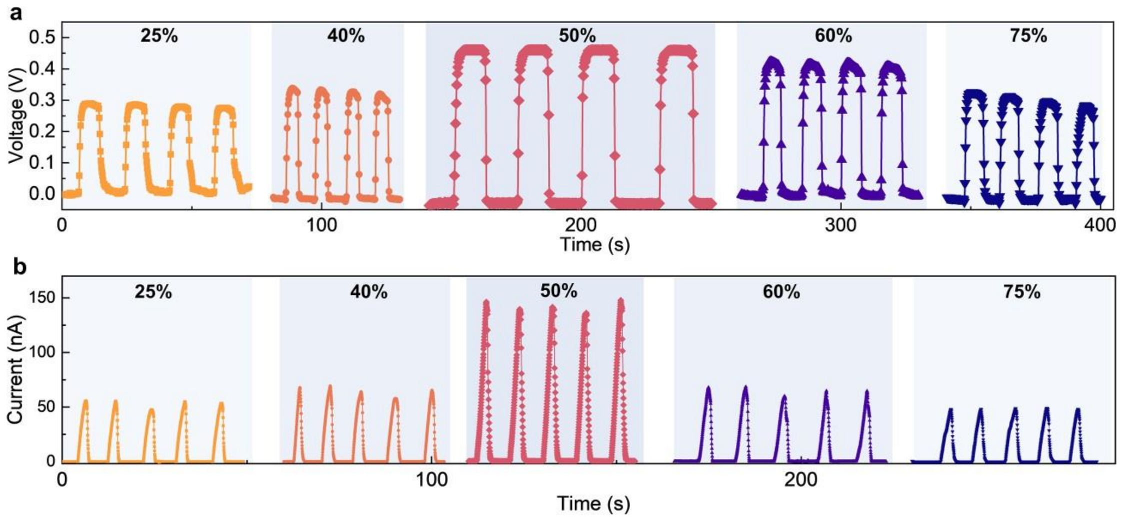

Fig. S27 Voltage and current responses of GZMEGs with different coverage areas, moisture at 90% RH was used for all humidification processes, and dry N_2 was used for all dehumidification processes

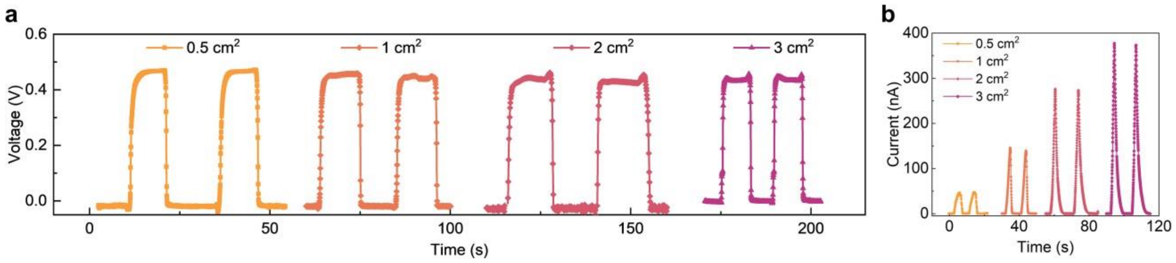

Fig. S28 Voltage and current responses of GZMEGs with different sizes, moisture at 90% RH was used for all humidification processes, and dry N_2 was used for all dehumidification processes

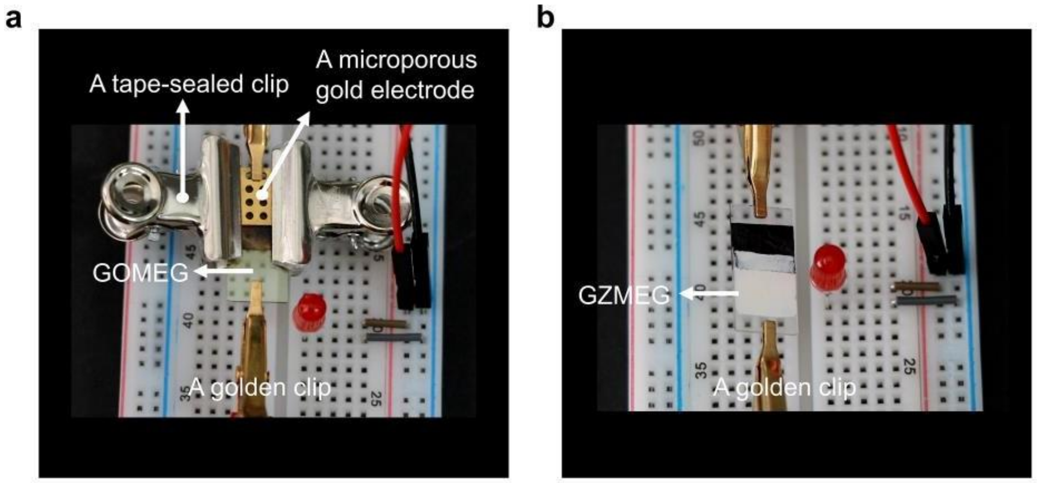

Fig. S29 Composition of GOMEG-controlled light-emitting diode (LED) and GZMEG-controlled LED, respectively

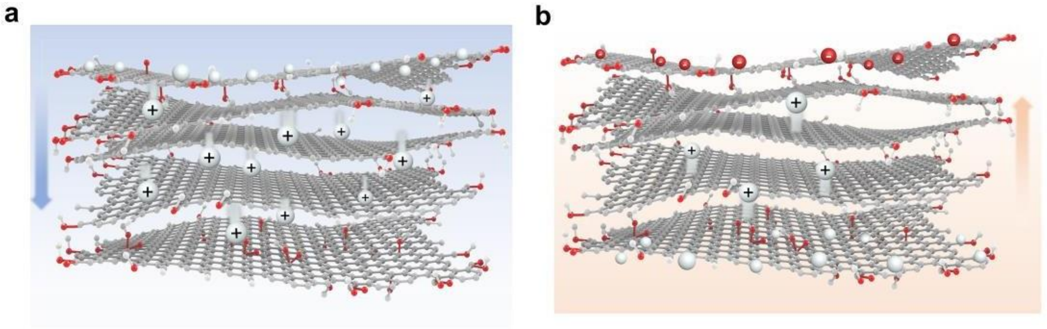

Fig. S30 Schematic diagram of the current and voltage output mechanism of GOMEG

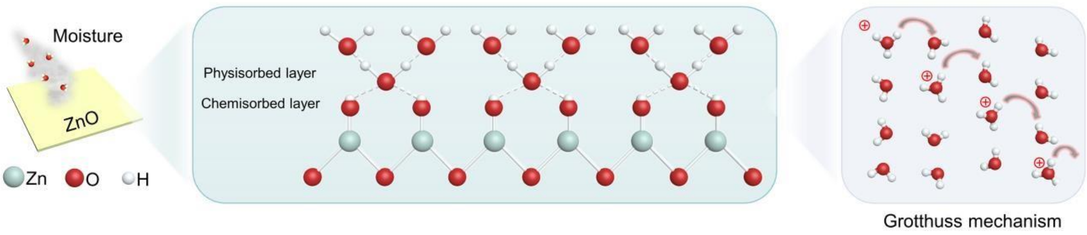

Fig. S31 Model diagram of ZnO adsorbing water molecules. Water molecules are adsorbed on the surface of ZnO by chemisorption and physical adsorption. The migration of H^+ between adjacent water molecules leads to charge transport according to the Grotthuss mechanism

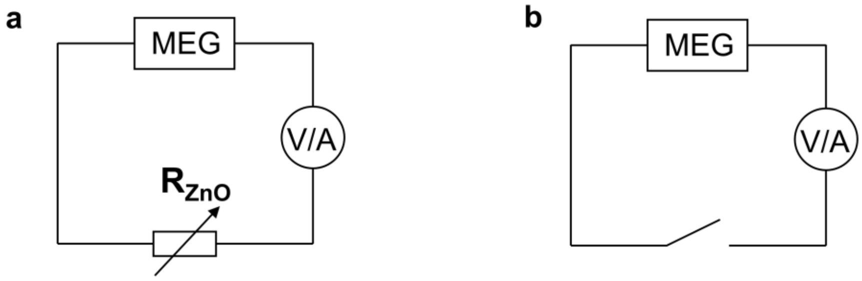

Fig. S32 Equivalent circuit diagram of GZMEG under low (a) and high (b) RH

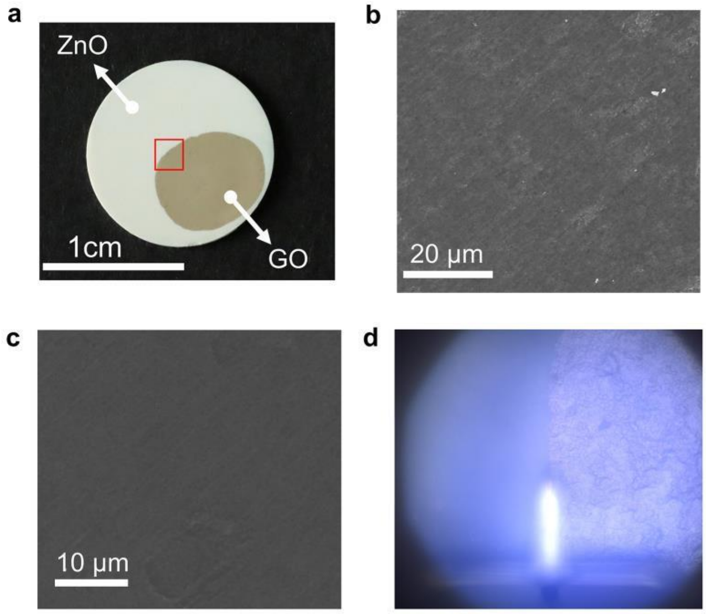

Fig. S33 KPFM test of GO-ZnO heterostructure. **a**) Photograph of a sample of the GO-ZnO heterostructure. **b**) SEM image of the ZnO side. **c**) SEM image of the GO side. **d**) Optical image of GO-ZnO heterostructures during KPFM testing

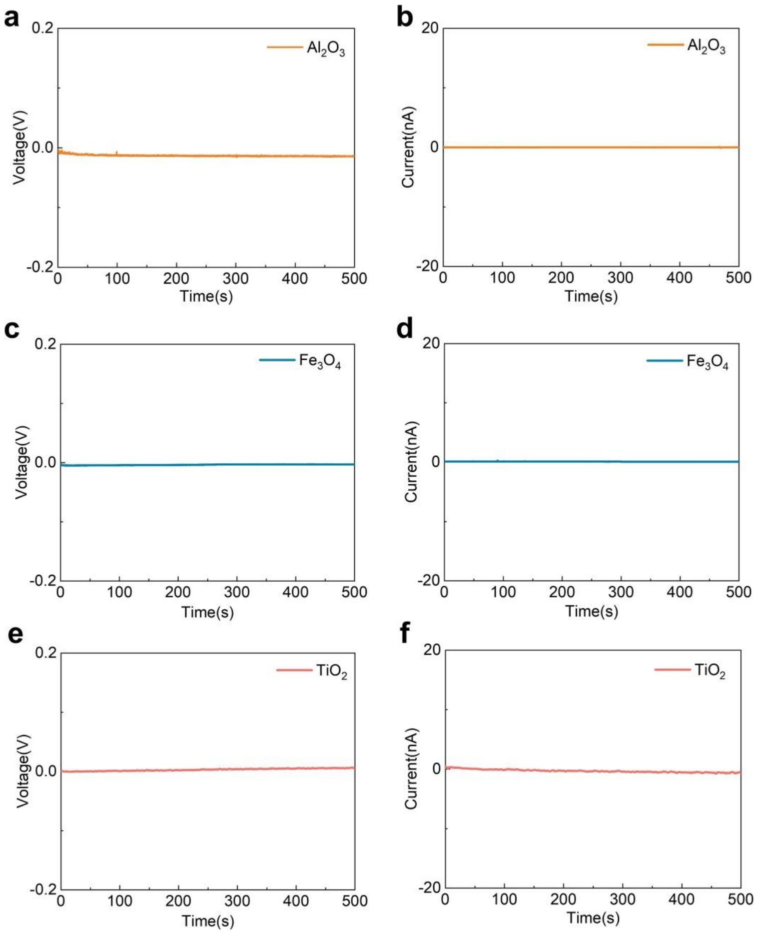

Fig. S34 Electrical output performance of moisture-sensitive oxides under RH of 90%. (**a, b**) Al_2O_3. (**c, d**) Fe_3O_4. (**e, f**) TiO_2

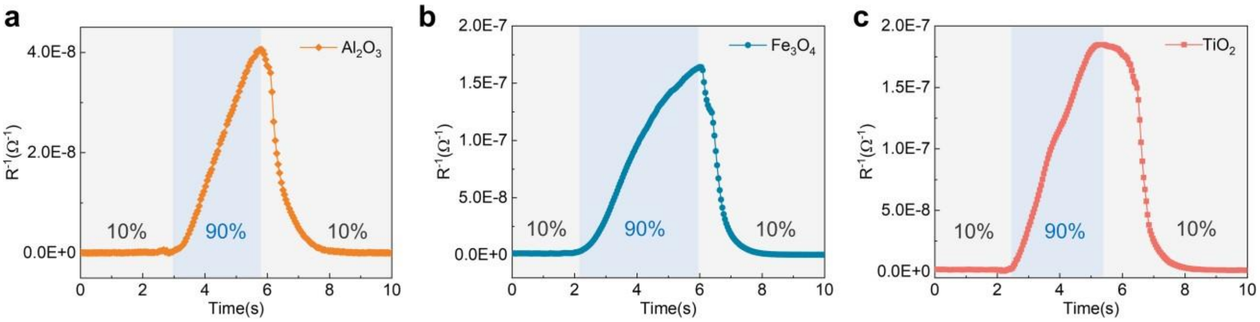

Fig. S35 Resistance changes of moisture-sensitive oxides under low (10%) and high (90%) RH. **a**) Al_2O_3. **b**) Fe_3O_4. **c**) TiO_2

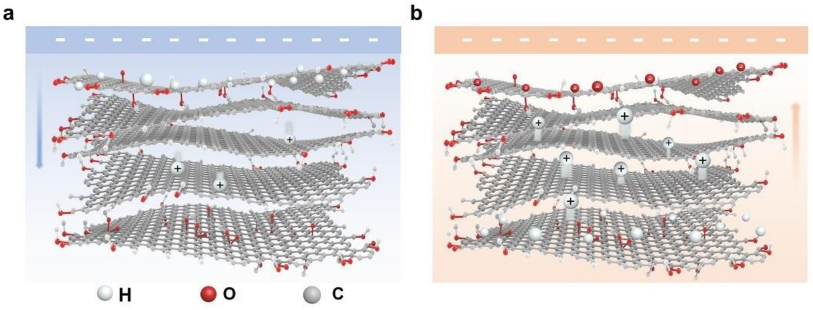

Fig. S36 Mechanistic diagram of MEG electrical output in the case of the moisture-sensitive oxide with negative zeta potential. **a**) Current output. **b**) Voltage output

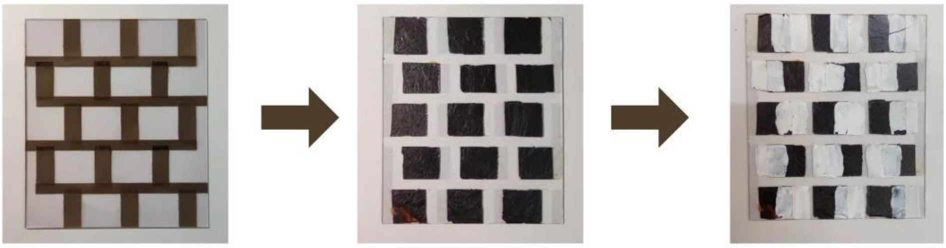

Fig. S37 The preparation process of the integrated GZMEG units by step-by-step screen printing

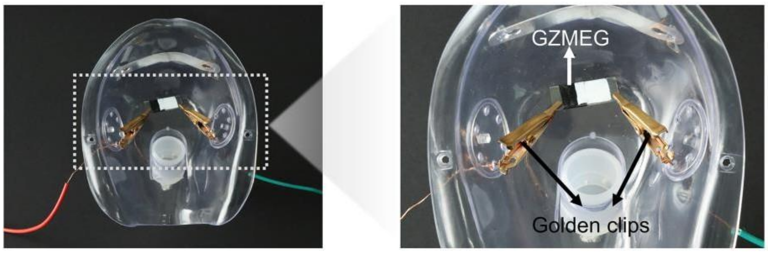

Fig. S38 The composition of a face mask with GZMEG as an HRM

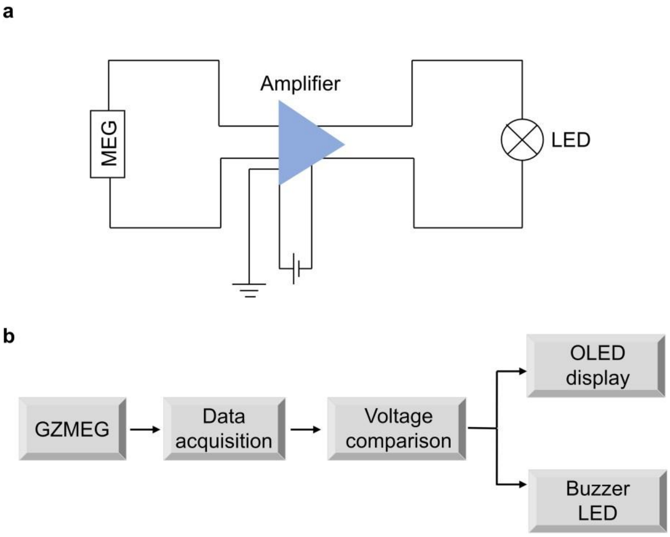

Fig. S39 Practical application of GZMEG as an HRM. **a**) Circuit diagram of the GZMEG-controlled respiratory indicator for real-time monitoring of respiration status. **b**) Schematic flow diagram of the apnea alarm controlled by GZMEG

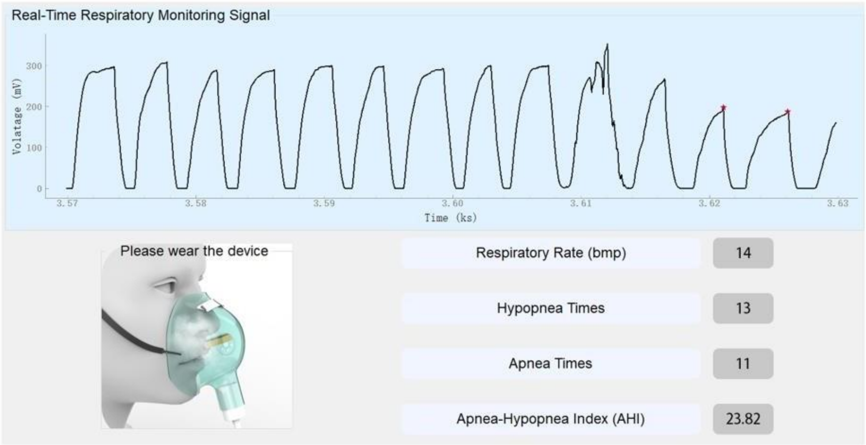

Fig. S40 Real-time monitoring of respiration by the OSAHS diagnostic system for one hour. The experiment was carried out on Apr. 25, 2022, in Beijing, China, with a 40% RH

Table S1 Comparison of voltage responses of different MEG systems

Material		Voltage variation range (mV)	Response time (s)	Average response rate (mV/s)	Reference
Polymer	Gradient PPy foam	59.4	4.62	12.9	[S5]
	Gradient PPy nanowire	69.4	0.199	343.7	[S6]
	Poly(4-styrensulfonic acid)membrane	914.8	2935.824	0.31	[S7]
	P(MEDSAH-co-AA)	345.2	2.433	141.9	[S8]
	Porous polydopamine	209.8	0.462	454.1	[S9]
	PVA nanofiber fabric	44.0	1032.9	0.04	[S10]
	PAN/PSSA nanofiber fabric	174.4	46.28	3.77	[S10]
	PSS-PVA	586.0	9.49	61.7	[S11]
Carbon-based materials	Porous carbon film	7.4	6.95	1.06	[S12]
	Carbon quantum dots	108.5	1207.72	0.09	[S13]
	Graphitic carbon	840.3	7.391	113.7	[S14]
	Oxygen-plasma treated amorphous carbon	124.9	12.52	9.98	[S15]
	Sucrose-sulfonated carbon	627.6	14.18	44.26	[S16]
Other materials	TiO_2 nanowire	461.9	192.86	2.4	[S17]
	MoS_2	18.8	83.52	0.23	[S18]
	Biological nanofiber	107.1	6.55	16.35	[S19]
	Gradient cellulose membrane	228.2	291.84	0.78	[S20]
	Paper	240.2	171.29	1.4	[S21]
	Cellulose acetate membranes	295.5	120.1	2.46	[S22]
	TiO_2 nanowire networks	359.7	7.73	46.5	[S23]
	Nanofiber fabric	1087.0	236.5	4.6	[S24]
	Biologic nanofibrils	67.1	1380.88	0.05	[S25]
Graphene	In-plane GO	64.2	6.44	9.97	[S26]
	GO quantum dots	130.8	2.72	48.1	[S27]
	GO Nanoribbon	41.7	1.58	26.4	[S28]
	GO fiber	353.1	6.65	53.1	[S29]
	GO foam	275.1	9.66	28.5	[S30]
	Gradient GO	886.5	483.44	1.83	[S31]
	GO film-1	426.9	129.59	3.29	[S1]

	GO film-2	683.3	423.07	1.62	[S2]
	GO film-3	304.8	173.14	1.76	[S3]
	GO film-4	194.8	110.78	1.76	[S32]
	GO film-5	216.9	1412.81	0.15	[S33]
**ME-MS Heterostructure**	**GO-ZnO**	**423**	**0.435**	**972.4**	**This work**

Table S2 Comparison of different MEG systems for respiratory monitoring

Material	ring voltage (mV)	ring time (s)	References
TiO_2 nanowire	~20	350	[S17]
Biological nanofiber	~40	35	[S19]
Porous polydopamine	~200	24	[S9]
MoS_2	16	20	[S18]
Cellulose acetate membranes	50	180	[S22]
Biologic nanofibrils	45	60	[S25]
sed moisture power generators	~400	40	[S34]
**GO-ZnO ME-MS heterostructure**	**~400**	**3600**	**This work**

Introduction to Movies

Movie S1: Water contact angles of ZnO flake and GO film. ZnO is super hydrophilic with a water contact angle close to 0°. GO is hydrophilic with a water contact angle of 51°.

Movie S2: Comparison of the responsiveness of GOMEG and GZMEG to moisture. This movie demonstrates the response to the moisture of LEDs controlled by GOMEG and GZMEG, respectively.

Movie S3: Real-time responses of a respiration indicator light controlled by GZMEG to human respiration. This movie shows the real-time response of the respiration indicator light controlled by GZMEG to breathing. The indicator light is off when inhaling, and the indicator light is on when exhaling. The breathing status is monitored in real-time by this method.

Movie S4: Apnea monitoring by GZMEG-controlled apnea alarm. This movie demonstrates the successful monitoring of apnea for more than 30 s by the alarm.

Movie S5: Judgment of respiratory status by OSAHS diagnostic system based on GZMEG. This movie displays that the system can distinguish between normal breathing, hypopnea, and apnea, and calculate the AHI to diagnose OSAHS.

Supplementary References

[S1] Xu T, Ding X, Shao C, Song L, Lin T, Gao X, et al. Electric power generation through the direct interaction of pristine graphene-oxide with water molecules. Small 2018, 14(14): e1704473. https://doi.org/10.1002/smll.201704473

[S2] Shao C, Gao J, Xu T, Ji B, Xiao Y, Gao C, et al. Wearable fiberform hygroelectric generator. Nano Energy 2018, 53: 698-705. https://doi.org/10.1016/j.nanoen.2018.09.043

[S3] Liang Y, Zhao F, Cheng Z, Deng Y, Xiao Y, Cheng H, et al. Electric power generation via asymmetric moisturizing of graphene oxide for flexible, printable and portable electronics. Energy & Environmental Science 2018, 11(7): 1730-1735. https://doi.org/10.1039/C8EE00671G

[S4] Cheng H, Huang Y, Zhao F, Yang C, Zhang P, Jiang L, et al. Spontaneous power source in ambient air of a well-directionally reduced graphene oxide bulk. Energy & Environmental Science 2018, 11(10): 2839-2845. https://doi.org/10.1039/C8EE01502C

[S5] Xue J, Zhao F, Hu C, Zhao Y, Luo H, Dai L, et al. Vapor-activated power generation on conductive polymer. Advanced Functional Materials 2016, 26(47): 8784-8792. https://doi.org/10.1002/adfm.201604188

[S6] Nie X, Ji B, Chen N, Liang Y, Han Q, Qu L. Gradient doped polymer nanowire for moistelectric nanogenerator. Nano Energy 2018, 46: 297-304. https://doi.org/10.1016/j.nanoen.2018.02.012

[S7] Xu T, Ding X, Huang Y, Shao C, Song L, Gao X, et al. An efficient polymer moist-electric generator. Energy & Environmental Science 2019, 12(3): 972-978. https://doi.org/10.1039/C9EE00252A

[S8] Long Y, He P, Shao Z, Li Z, Kim H, Yao AM, et al. Moisture-induced autonomous surface potential oscillations for energy harvesting. Nature Communications 2021, 12(1): 5287. https://doi.org/10.1038/s41467-021-25554-y

[S9] Li L, Chen Z, Hao M, Wang S, Sun F, Zhao Z, et al. Moisture-driven power generation for multifunctional flexible sensing systems. Nano Letters 2019, 19(8): 5544-5552. https://doi.org/10.1021/acs.nanolett.9b02081

[S10] Sun Z, Feng L, Xiong C, He X, Wang L, Qin X, et al. Electrospun nanofiber fabric: an efficient, breathable and wearable moist-electric generator. Journal of Materials Chemistry A 2021, 9(11): 7085-7093. https://doi.org/10.1039/D0TA11974A

[S11] Wang H, Cheng H, Huang Y, Yang C, Wang D, Li C, et al. Transparent, self-healing, arbitrary tailorable moist-electric film generator. Nano Energy 2020, 67: 104238. https://doi.org/10.1016/j.nanoen.2019.104238

[S12] Liu K, Yang P, Li S, Li J, Ding T, Xue G, et al. Induced potential in porous carbon films through water vapor absorption. Angewandte Chemie-International Edition 2016, 55(28): 8003-8007. https://doi.org/10.1002/anie.201602708

[S13] Li Q, Zhou M, Yang Q, Yang M, Wu Q, Zhang Z, et al. Flexible carbon dots composite paper for electricity generation from water vapor absorption. Journal of Materials Chemistry A 2018, 6(23): 10639-10643. https://doi.org/10.1039/C8TA02505C

[S14] Lee S, Eun J, Jeon S. Facile fabrication of a highly efficient moisture-driven power generator using laser-induced graphitization under ambient conditions. Nano Energy 2020, 68: 104364. https://doi.org/10.1016/j.nanoen.2019.104364

[S15] Tao Y, Wang Z, Xu H, Ding W, Zhao X, Lin Y, et al. Moisture-powered memristor with interfacial oxygen migration for power-free reading of multiple memory states. Nano Energy 2020, 71: 104628. https://doi.org/10.1016/j.nanoen.2020.104628

[S16] Gao K, Sun J, Lin X, Li Y, Sun X, Chen N, et al. High-performance flexible and integratable MEG devices from sulfonated carbon solid acids containing strong Brønsted acid sites. Journal of Materials Chemistry A 2021, 9(43): 24488-24494. https://doi.org/10.1039/D1TA06757E

[S17] Shen D, Xiao M, Zou G, Liu L, Duley WW, Zhou YN. Self-powered wearable electronics based on moisture enabled electricity generation. Advanced materials 2018, 30(18): e1705925. https://doi.org/10.1002/adma.201705925

[S18] He D, Yang Y, Zhou Y, Wan J, Wang H, Fan X, et al. Electricity generation from phase-engineered flexible MoS2 nanosheets under moisture. Nano Energy 2021, 81: 105630. https://doi.org/10.1016/j.nanoen.2020.105630

[S19] Li M, Zong L, Yang W, Li X, You J, Wu X, et al. Biological nanofibrous generator for electricity harvest from moist air flow. Advanced Functional Materials 2019, 29(32): 1901798. https://doi.org/10.1002/adfm.201901798

[S20] Lee S, Jang H, Lee H, Yoon D, Jeon S. Direct fabrication of a moisture-driven power generator by laser-induced graphitization with a gradual defocusing method. ACS Applied Materials & Interfaces 2019, 11(30): 26970-26975. https://doi.org/10.1021/acsami.9b08056

[S21] Gao X, Xu T, Shao C, Han Y, Lu B, Zhang Z, et al. Electric power generation using paper materials. Journal of Materials Chemistry A 2019, 7(36): 20574-20578. https://doi.org/10.1039/C9TA08264F

[S22] Lyu Q, Peng B, Xie Z, Du S, Zhang L, Zhu J. Moist-induced electricity generation by electrospun cellulose acetate membranes with optimized porous structures. ACS Applied Materials & interfaces 2020, 12(51): 57373-57381. https://doi.org/10.1021/acsami.0c17931

[S23] Shen D, Xiao M, Xiao Y, Zou G, Hu L, Zhao B, et al. Self-powered, rapid-response, and highly flexible humidity sensors based on moisture-dependent voltage generation. ACS Applied Materials & Interfaces 2019, 11(15): 14249-14255. https://doi.org/10.1021/acsami.9b01523

[S24] Sun Z, Feng L, Wen X, Wang L, Qin X, Yu J. Nanofiber fabric based ion-gradient-enhanced moist-electric generator with a sustained voltage output of 1.1 volts. Mater Horiz 2021, 8(8): 2303-2309. https://doi.org/10.1039/D1MH00565K

[S25] Yang W, Li X, Han X, Zhang W, Wang Z, Ma X, et al. Asymmetric ionic aerogel of biologic nanofibrils for harvesting electricity from moisture. Nano Energy 2020, 71: 104610. https://doi.org/10.1016/j.nanoen.2020.104610

[S26] Cheng H, Huang Y, Qu L, Cheng Q, Shi G, Jiang L. Flexible in-plane graphene oxide moisture- electric converter for touchless interactive panel. Nano Energy 2018, 45: 37-43. https://doi.org/10.1016/j.nanoen.2017.12.033

[S27] Huang Y, Cheng H, Shi G, Qu L. Highly Efficient Moisture-Triggered Nanogenerator Based on Graphene Quantum Dots. ACS applied materials & interfaces 2017, 9(44): 38170-38175. https://doi.org/10.1021/acsami.7b12542

[S28] Zhao F, Wang L, Zhao Y, Qu L, Dai L. Graphene Oxide Nanoribbon Assembly toward Moisture- Powered Information Storage. Advanced materials 2017, 29(3). https://doi.org/10.1002/adma.201604972

[S29] Liang Y, Zhao F, Cheng Z, Zhou Q, Shao H, Jiang L, et al. Self-powered wearable graphene fiber for information expression. Nano Energy 2017, 32: 329-335. https://doi.org/10.1016/j.nanoen.2016.12.062

[S30] Zhao F, Liang Y, Cheng H, Jiang L, Qu L. Highly efficient moisture-enabled electricity generation from graphene oxide frameworks. Energy & Environmental Science 2016, 9(3): 912- 916. https://doi.org/10.1039/C5EE03701H

[S31] Huang Y, Cheng H, Yang C, Zhang P, Liao Q, Yao H, et al. Interface-mediated hygroelectric generator with an output voltage approaching 1.5 volts. Nature communications 2018, 9(1): 4166. https://doi.org/10.1038/s41467-018-06633-z

[S32] Yang C, Huang Y, Cheng H, Jiang L, Qu L. Rollable, Stretchable, and Reconfigurable Graphene Hygroelectric Generators. Advanced materials 2019, 31(2): e1805705. https://doi.org/10.1002/adma.201805705

[S33] Han Y, Lu B, Shao C, Xu T, Liu Q, Liang Y, et al. A hygroelectric power generator with energy self-storage. Chemical Engineering Journal 2020, 384: 123366. https://doi.org/10.1016/j.cej.2019.123366

[S34] He W, Wang H, Huang Y, He T, Chi F, Cheng H, et al. Textile-based moisture power generator with dual asymmetric structure and high flexibility for wearable applications. Nano Energy 2022, 95: 107017.https://doi.org/10.1016/j.nanoen.2022.107017
